# *Encephalartos villosus* Lem. Displays a Strong In Vivo and In Vitro Antifungal Potential against *Candida glabrata* Clinical Isolates

**DOI:** 10.3390/jof8050521

**Published:** 2022-05-18

**Authors:** Moneerah J. Alqahtani, Engy Elekhnawy, Walaa A. Negm, Sebaey Mahgoub, Ismail A. Hussein

**Affiliations:** 1 Department of Pharmacognosy, College of Pharmacy, King Saud University, P.O. Box 2457, Riyadh 11451, Saudi Arabia; mjalqahtani@ksu.edu.sa; 2 Department of BioMolecular Sciences, Division of Pharmacognosy, School of Pharmacy, University of Mississippi, Oxford, MS 38677, USA; 3 Pharmaceutical Microbiology Department, Faculty of Pharmacy, Tanta University, Tanta 31527, Egypt; 4 Department of Pharmacognosy, Faculty of Pharmacy, Tanta University, Tanta 31527, Egypt; 5 Food Analysis Laboratory, Ministry of Health, Zagazig 44511, Egypt; dr_semahgoub@yahoo.com; 6 Department of Pharmacognosy and Medicinal Plants, Faculty of Pharmacy (Boys), Al-Azhar University, Cairo 11884, Egypt; ismaila.hussein@azhar.edu.eg

**Keywords:** efflux, kidney, LC–MS/MS, qRT-PCR, survival curve, TNF-α

## Abstract

Recently, *Candida glabrata* has been recognized as one of the most common fungal species that is highly associated with invasive candidiasis. Its spread could be attributed to its increasing resistance to antifungal drugs. Thus, there is a high need for safer and more efficient therapeutic alternatives such as plant extracts. Here, we investigated the antifungal potential of *Encephalartos villosus* leaves methanol extract (EVME) against *C. glabrata* clinical isolates. Tentative phytochemical identification of 51 metabolites was conducted in EVME using LC–MS/MS. EVME demonstrated antifungal activity with minimum inhibitory concentrations that ranged from 32 to 256 µg/mL. The mechanism of the antifungal action was studied by investigating the impact of EVME on nucleotide leakage. Additionally, a sorbitol bioassay was performed, and we found that EVME affected the fungal cell wall. In addition, the effect of EVME was elucidated on the efflux activity of *C. glabrata* isolates using acridine orange assay and quantitative real-time PCR. EVME resulted in downregulation of the expression of the efflux pump genes *CDR1*, *CDR2*, and *ERG11* in the tested isolates with percentages of 33.33%, 41.67%, and 33.33%, respectively. Moreover, we investigated the in vivo antifungal activity of EVME using a murine model with systemic infection. The fungal burden was determined in the kidney tissues. Histological and immunohistochemical studies were carried out to investigate the effect of EVME. We noticed that EVME reduced the congestion of the glomeruli and tubules of the kidney tissues of the rats infected with *C. glabrata*. Furthermore, it decreased both the proinflammatory cytokine tumor necrosis factor-alpha and the abnormal collagen fibers. Our results reveal, for the first time, the potential in vitro (by inhibition of the efflux activity) and in vivo (by decreasing the congestion and inflammation of the kidney tissues) antifungal activity of EVME against *C. glabrata* isolates.

## 1. Introduction

Globally, fungal infections have been increasing dramatically over the past few decades. Several fungi, such as *Candida*, *Aspergillus*, and *Cryptococcus*, commonly cause human infections. *Candida* spp. is regarded as the most common cause of nosocomial infections caused by fungi. Among all hospitalized patients, immunocompromised patients are the most affected by fungal infections, with high morbidity and mortality rates [1].

*Candida glabrata* is a common fungal pathogen in humans. It has become the second worldwide cause of candidiasis after *C. albicans*. It is a commensal fungus of mucosal surfaces such as the oropharyngeal cavity, vagina, and gastrointestinal tract. However, it is also an opportunistic pathogen capable of causing superficial and disseminated life-threatening infections such as candidemia [2].

The fungus *C. glabrata* is a haploid pathogenic microorganism, and it is intrinsically less sensitive to azole antifungals. Thus, invasive infections are highly associated with a relatively high mortality rate, especially in immunocompromised and elderly patients. The infections caused by *C. glabrata* are especially difficult to treat owing to its high antifungal resistance to many antifungals such as echinocandins and azoles [2]. Its spreading resistance to many antifungal drugs is a surprising and alarming issue.

Efflux is one of the major mechanisms of resistance of *C. glabrata* to antifungal agents. It is mainly mediated by major facilitators superfamily as well as ATP-binding cassette transporters pumps. These pumps are encoded by *MDR* and *CDR* genes, respectively [3]. The rapid efflux in the resistant isolates can prevent the antifungal drug from being accumulated in the fungal cells to lethal levels. Thus, many resistant *C. glabrata* clinical isolates are able to display a transcriptional activation of the genes encoding efflux pumps. These isolates exhibit reduced intracellular accumulation of the antifungal drugs, thereby confirming the major role of efflux pumps in antifungal drug extrusion and resistance [4].

This fact necessitates developing novel antifungal therapies. Therefore, many researchers have investigated the efficacy of natural and synthetic agents as antifungal agents [5]. Natural agents, particularly plants, are rich sources for a wide variety of bioactive secondary metabolites such as saponins, tannins, alkaloids, terpenoids, and flavonoids [6,7]. These compounds have been reported to possess in vitro antifungal activity. In addition, plants have many advantages for treating fungal infections, as they are safe, effective, available, and cheap [8]. Thus, the antimicrobial activities of plant extracts are the basis of various applications, such as pharmaceuticals, food preservation, alternative medicine, and natural therapies [9].

*Encephalartos* (Family Zamiaceae), the second-largest extant genus of the Cycadales, is most frequently cultivated in Africa, south of the Sahara, with roughly 66 species. The tropical regions of central and east Africa have many species, but South Africa has more than half of them. They are endemism-rich and occupy various climate regimes and habitats [10,11]. 

*Encephalartos villosus* Lem. is one of the most popular decorative dwarf cycads. It is often known as poor man’s cycad. *E. villosus* prefers the shade and has gracefully spreading leaves with glossy dark green leaflets. This species overgrows and will mature into a large plant in 5 to 8 years. A former phytochemical study of *E. villosus* leaves revealed the isolation of four flavone glycosides luteolin-7-rutinoside, luteolin-7-glucoside, luteolin-7-rhamnoside, and apigenin-7-glucoside and two new illudalane sesquiterpenes, named encephaldiene 1 and encephaldiene 2. The plant *E. villosus* exhibited antifungal *Aspergillus fumigatus* and antibacterial action against *Streptococcus pneumoniae*, *Bacillus subtilis,* and *Escherichia coli* [12].

In the current study, we explored the antifungal efficacy of EVME against *C. glabrata* clinical isolates both in vitro and in vivo. In addition, the phytochemical profile of EVME was investigated using LC–MS.

## 2. Materials and Methods

### 2.1. Chemicals

All media were obtained from Oxoid, UK. Chemicals and solvents were obtained from Merck, NJ, USA.

### 2.2. Plant Materials

*Encephalartos villosus* Lem. leaves were collected from Al Orman Botanical Garden on 15 July 2021. The plant was recognized by Dr. Esraa Ammar, Plant Ecology, Tanta University. A voucher sample (PG-G-106-E) was deposited at the Herbarium of the Faculty of Science, Tanta University. The plant was dried at room temperature for ten days, then in an oven at 40 °C for two days, and then ground to a coarse, powdered form (particle size 2000/335). The powder (300 g) was extracted by methanol (4 L × three times) at two-day intervals using the cold maceration method at room temperature to yield 31.39 g of EVME.

### 2.3. Animals

A total of 30 male albino rats, which weighed 120–150 g and were aged eight weeks old, were obtained from the Animal House of the Faculty of Veterinary Medicine, Cairo University, Egypt. They were allowed to acclimate for seven days under the standard environmental conditions. In addition, they were maintained on a regular feed and water ad libitum and allowed to adapt for one week. The authorized standards of using laboratory animals were followed according to the Faculty of Pharmacy Research Ethical Committee (Tanta University), with the code number (TP/RE/04-22-P-008).

### 2.4. LC–MS/MS for Metabolite Profiling

The liquid chromatography–electrospray ionization–tandem mass spectrometry (LC–ESI–MS/MS) analysis was carried out at the Children’s Cancer Hospital’s Proteomics and Metabolomics Unit (57357). Adopting the criteria previously described [13,14]. Secondary metabolites of the EVME were analyzed using an ExionLC-High flow L.C., Sciex^®^, Framingham, MA, USA, Ultra performance liquid chromatography (UPLC) analytical technique, combined with a Triple time-of-flight (TOF) 5600+(Sciex^®^) for IDA acquisition and Analyst TF 1.7.1 (Sciex^®^) for LC–Triple TOF control. After the filtration process via an in-line filter disk (0.5 μm × 3.0 mm, Phenomenex^®^, Torrance, CA, USA), 1 μg/mL of EVME was injected into an X Select HSS T3 XP ultra-high-performance liquid chromatography (UHPLC) column (2.5 μm, 2.1 × 150 mm, Waters^®^, Milford, MA, USA) at 40 °C and eluted using buffer systems of 1% methanol in 5 mM ammonium formate buffer at pH 3 and pH 8 as solvent A (for positive mode MS analysis) and B (for negative mode MS analysis), respectively, and 100% of acetonitrile as solvent C at 0.3 mL/min flow rate. Gradient mobile phase mixtures were composed of 90% solvent A or B and 10% of solvent C; they were injected for 20 min, then reversed into 10 % of solvent A or B to 90% of solvent C for the next 5 min, and finalized by loading of the starting mixture for the last 3 min. The resultant total ion chromatogram (TIC) was used for master view peaks extraction with a signal-to-noise ratio greater than 5 (non-targeted analysis) and more than three features’ intensities of the sample-to-blank ratio. Data interpretation was accomplished by applying a Reifycs Abf (Analysis Base File) Converter (Reifycs^®^, Tokyo, Japan) for Wiff file conversion and MS-DIAL 4.6 (RIKEN^®^, Tokyo, Japan).

To identify chemicals, PeakView^TM^ software was used to compare retention times and *m*/*z* values obtained with MS and MS^2^. The XIC Manager in PeakView^TM^ software was used to calculate peak area values. Extracted ion chromatograms (XICs) for each targeted analyte were automatically created and compared with a user-defined threshold [15].

### 2.5. In Vitro Antifungal Activity of EVME

#### 2.5.1. Fungi

*C. glabrata* clinical isolates were obtained from different specimens, including blood, sputum, and urine, from the clinical laboratories of Tanta University Hospitals, Tanta, Egypt. These specimens were obtained from patients for routine diagnosis, and their data were obscured. First, the specimens were inoculated onto Sabouraud dextrose agar (SDA) plates, and the plates were incubated at 37 °C with a relative humidity of 97%. *Candida* isolates were identified via microscopical examination, as well as examination using matrix-assisted laser desorption ionization–time of flight (MALDI–TOF) (bioMerieux, France).

#### 2.5.2. Antifungal Susceptibility

Agar well dilution method was utilized to investigate the antifungal activity of EVME, as previously described [16,17]. After spreading the fungal suspension on the surface of SDA plates, cups were punched off the agar. Each plate contained three wells—one with EVME at a concentration of 1000 µg/mL, a second well considered as a negative control (10% dimethyl sulfoxide or DMSO), and a third well, which was a positive control (fluconazole). The plates were then incubated overnight at 37 °C and inspected for inhibition zone formation.

#### 2.5.3. Determination of Minimum Inhibitory Concentrations (MICs)

MICs were determined using the broth microdilution method in microtitration plates [17]. In brief, after centrifugation of the fungal suspensions and washing of the obtained pellets, they were resuspended in Roswell Park Memorial Institute 1640 (RPMI 1640) to obtain a fungal concentration of 1× 10^3^ CFU/mL, and 50 µL was added to each well. EVME was serially diluted from 1024 to 1 µg/mL in the wells of the microtitration plates. In addition, each plate had a well that contained fungi only (positive control) and another well that contained RPMI 1640 only, without fungi (negative control). The plates were then incubated overnight at 37 °C. MIC values of EVME were recorded as the lowest concentrations, which inhibited the growth of *C. glabrata* isolates.

#### 2.5.4. Time–Kill Curve

It was carried out in order to determine the time–kill kinetics for *C. glabrata* isolates [18]. In brief, overnight cultures of *C. glabrata* isolates in brain–heart infusion (BHI) broth were standardized to 10^6^ CFU/mL, and different concentrations of EVME (0.5× MIC, 1× MIC, 2× MIC, 4× MIC, and 8× MIC values) were added. Then, the optical density (OD) values were recorded at 530 nm at different time intervals (1, 2, 3, 4, 8, and 24 h of incubation) at 37 °C. Finally, graphs of OD values against incubation time were constructed.

#### 2.5.5. Nucleotide Leakage

The release of nucleotides from the fungal cells was evaluated as described before [18]. Briefly, overnight cultures of *C. glabrata* isolates were incubated with EVME (0.5× MIC). Then, OD values at 260 nm were recorded at different time points (0, 1, 2, 3, 4, and 5 h) using a UV–Vis spectrophotometer (SHIMADZU, Japan). Positive control (using fluconazole) and negative control (fungal isolates in BHI) were used. 

#### 2.5.6. Sorbitol Protection Assay

This test was carried out to determine the effect of EVME on the fungal cell wall [18]. EVME was serially diluted (starting from a concentration of 1025 µg/mL) in microtitration plates containing BHI broth and 0.8 M sorbitol. Positive and negative controls were included in each plate as previously described. The microtitration plates were incubated at 37 °C for 24 h, and the lowest concentrations of EVME inhibited the fungal growth were recorded.

#### 2.5.7. Phenotypic Detection of Efflux Pump Inhibition

It was performed as described previously [4]. *C. glabrata* isolates were overnight grown on SDA then fungal suspensions (0.5 McFarland) were prepared. Using sterile cotton swabs, the fungal suspensions, before and after treatment with EVME, were swabbed onto SDA containing different concentrations of acridine orange (1, 5, 10, and 20 mg/mL) from the center of the plate toward its periphery. With the cartwheel design, the swabbed SDA plates were incubated for 24 h at 37 °C, and the fluorescence of the fungal swabs was visualized using a UV transilluminator (Kowell, Spain). The lowest concentration of acridine orange that produced green fluorescence was recorded for each *C. glabrata* isolate. Isolates that did not produce any fluorescence at the tested concentrations of acridine orange were considered to have strong efflux activity. In addition, isolates that produced fluorescence at a high concentration of acridine orange (20 mg/mL) were considered to have moderate efflux activity. On the other hand, isolates that emitted fluorescence at 5 and 10 mg/mL were considered to have weak efflux activity. Isolates that produced fluorescence at 1 mg/mL were considered to lack efflux activity.

#### 2.5.8. Quantitative Real-Time PCR (qRT-PCR) for Detection of the Expression of the Efflux Pump Genes

In order to determine the influence of EVME on the gene expression of the genes that encode efflux pumps (*CDR1*, *CDR2*, and *ERG11*), RT-PCR was used. After extracting the fungal RNA using the RNeasy Mini Kit (QIAGEN, Germany), cDNA was obtained using the SensiFAST™ cDNA Kit (Bioline, UK). qRT-PCR was accomplished using SensiFAST™ SYBR Green PCR Master Mix (Bioline, UK), as described by the manufacturer. The utilized primers are listed in Table S1, using the *URA3* gene as a reference gene [4]. Gene expression in *C. glabrata* isolates before treatment was considered to be one.

### 2.6. In Vivo Antifungal Activity of EVME

#### 2.6.1. Experimental Protocol

All animals were subjected to immunosuppression using corticosteroid injection. Then, they were randomly grouped into the following three groups [19]:

Group I (*C. glabrata* group): Ten rats each received a fungal suspension of *C. glabrata* (0.1 mL) once via intravenous (IV) injection in the tail vein;

Group II (fluconazole treated group): Ten rats each received a fungal suspension of *C. glabrata* (0.1 mL) once via IV injection in the tail vein. After that, this group received fluconazole (10 mg/kg) via intraperitoneal (IP) injection for one week;

Group III (EVME treated group): Ten rats each received a fungal suspension of *C. glabrata* (0.1 mL) once via IV injection in the tail vein. Then, this group received 50 mg/kg EVME via IP injection once per day for one week.

Rats were monitored for 14 days after infection to determine the survival rate. After being anesthetized, the rats were sacrificed, blood samples were collected once after scarification, and kidney tissues were acquired for histological and immunohistochemical studies. Moreover, the fungal burden in the kidney tissues was calculated as the number of CFU/g kidney tissues.

#### 2.6.2. Kidney Function Tests

Serum (collected after blood clotting) was used to assess kidney function by detecting blood urea and serum creatinine levels.

#### 2.6.3. Histological Assessment

Samples of kidney tissues were collected from each rat, then fixed with formalin (10%) for 72 h to be histopathologically examined. The tissue was dehydrated, embedded in paraffin wax, sectioned (3 μm), and stained with hematoxylin and eosin (H&E) [20] and Masson’s trichrome stains. Finally, photos were captured using a digital camera [13,14]. 

#### 2.6.4. Immunohistochemical Studies

Immunostaining was carried out for tumor necrosis factor-alpha (TNF-α) using a TNF-α polyclonal antibody (PA1- 40281, Invitrogen, Massachusetts, USA). Immunostaining of TNF-α was examined using a light microscope at a magnification of ×100. TNF-α staining was regarded as a bad indication. We graded the staining strength into (0) negative, meaning an absence of immunoreactive cells; (1) weak, meaning the presence of 1–10% immunoreactive cells; (2) moderate, meaning the presence of 11–50% immunoreactive cells; and (3) strong meaning presence of more than 50% immunoreactive cells [21].

### 2.7. Statistical Analysis

Results are shown as mean ± standard deviation, as all the performed tests were carried out in triplicate. The differences among the tested groups were evaluated using a one-way analysis of variance (ANOVA), followed by a post hoc test (Tukey). The difference was considered to be significant when *p* < 0.05. Furthermore, the Kaplan–Meier survival curve was utilized to calculate the survival of rats using Prism version 8 (GraphPad Software, Inc., California, USA).

## 3. Results

### 3.1. Phytochemical Profiling of EVME

**Figure 1:**
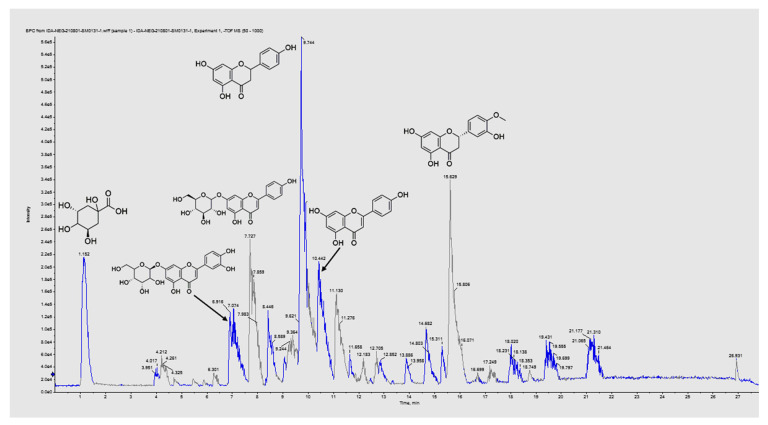
The total ion chromatogram of EVME presented the major identified metabolites (D-(−)-quinic acid, luteolin-7-*O*-glucoside, apigenin-7-*O*-glucoside, naringenin, apigenin, and hesperetin according to the retention time) via LC–ESI–MS/MS in negative ion mode.

LC–ESI–MS/MS analysis of EVME (negative ESI mode) tentatively identified 51 secondary metabolites compounds for the first time. The identified metabolites were flavonoid and glycosides, phenolic, carboxylic acids, fatty acids, etc. Table 1 shows the LC–ESI–MS/MS analysis of EVME metabolites, and Figure 1 presents the total ion chromatogram (TIC) of EVME in negative mode, while Figure S1 shows MS2 spectral fragmentation of all identified metabolites.

#### 3.1.1. Identification of Carboxylic, Phenolic, and Fatty Acids

According to our LC–MS/MS analysis, EVME exhibited variety of phenolic compounds such as D-(+)-malic acid (*m*/*z* 133.02), (−)-shikimic acid (*m*/*z* 173.04), maleic acid (*m*/*z* 115.0), D-(−)-quinic acid (*m*/*z* 191.05), 3,4-dihydroxybenzoic acid (*m*/*z* 153.01), caffeic acid (*m*/*z* 179.05), 3-(4-hydroxy-3-methoxy phenyl) prop-2-enoic acid (*m*/*z* 193.05), salicylic acid (*m*/*z* 137.02), (+/−)-*cis,trans*-abscisic acid (*m*/*z* 263.12), 5-methoxy salicylic acid (*m*/*z* 167.03), rosmarinic acid (*m*/*z* 359.11), and isocitrate (*m*/*z* 190.95). D-(−)-quinic acid was the major detected carboxylic acid according to peak height and area measurements.

The detected fatty acids were 3-hydroxy-3- methyl glutaric acid, 2-Isopropyl malic acid, citraconic acid, citramalate, and gamma-Linolenic acid [M-H] at *m*/*z* values of 161.04, 175.05, 129.01, 147.06, and 277.14, respectively.

#### 3.1.2. Identification of Flavonoid Derivatives

Flavonoid aglycones and their glycosides resemble most of the compounds detected in EVME. Different subclasses such as flavones, flavanones, flavonols, flavanonols, as well as isoflavonoids, biflavonoids, and polyflavonoids, were found. 

Apigenin and luteolin flavone aglycones were established by the corresponding base peak and the fragmentation pattern for each compound. In the negative ion mode analysis, the MS/MS fragment at *m*/*z* 151.00 is characteristic for 5, 7-dihydroxy flavonoids, which were detected in apigenin and luteolin. 

The [M-H]^−^ ion of luteolin-7-*O*-glucoside and luteolin-3’, 7-di-*O*-glucoside at *m*/*z* 447.09 and 609.13, with loss of neutral ions of one or two hexose or glucose molecules (324 Da), was detected by fragment ion of aglycone at *m*/*z* 285.03. The [M-H]^−^ ion of baicalein-7-*O*-glucuronide was observed at *m*/*z* 445.07 and the baicalein ion fragment presented at *m*/*z* 269.02 due to glucuronide moiety loss. Rhoifolin (apigenin 7-*O*-neohesperidoside) ion fragment was found at *m*/*z* 577.13.

The tentatively identified flavanone aglycones were naringenin and hesperetin, with [M-H]^−^ ion at *m*/*z* 271.09 and 301.10, respectively. They are the most highly representative individual compounds in EVME. Hesperetin showed [M-H]^−^ ions at *m*/*z* 301.1 and MS/MS fragment ions at 151 [C_7_H_4_O_4_]-H-. The pseudo molecular ions of 3’,4’,5,7-Tetrahydroxy flavanone, naringenin-7-*O*-glucoside, and isookanin-7-glucoside were detected at *m*/*z* 287.05, 433.20, and 449.09, respectively.

Kaempferol and its glycosides represent the majority of the flavonol subclass. In the negative mode analysis, the MS/MS fragment at *m*/*z* 151 confirmed the 5,7-dihydroxy pattern of the aglycones. The loss of 162 Daltons identified hexose or glucose motifs, the loss of 146 Da identified the rhamnose moiety, and the loss of rutinoside or neohesperoside (308 Da). LC–ESI/MS showed flavonol metabolites such as kaempferol-3-*O*-α-L-rhamnoside (*m*/*z* 431.18), Kaempferol-7-neohesperidoside (*m*/*z* 593.14) kaempferol-3-glucuronide (*m*/*z* 461.10), kaempferol-3-*O*- α -L-arabinoside (*m*/*z* 417.11), 3’-methoxy-4’,5,7-trihydroxy flavonol (*m*/*z* 315.05), hyperoside (*m*/*z* 463.08), 3,5,7-trihydroxy-4’-methoxyflavone (*m*/*z* 299.09), and 4’,5,7-trihydroxy flavonol (*m*/*z* 285.11).

The pseudo-molecular ions of isorhamnetin-3-*O*-rutinoside and isorhamnetin-3-*O*-glucoside were noted at *m*/*z* 623.12 and 477.10, respectively. Isorhamnetin-3-*O*-glucoside presented MS/MS fragments at *m*/*z* 315.04 [M-H-glc]-, 299.01 [M-H-glc-H_2_O]-, and 285.03 [M-H-glc-OCH_3_]-, while isorhamnetin-3-*O*-rutinoside observed ions due to neutral loss of CO_2_ (44 Da) at *m*/*z* 579.00, and loss of rutinoside (308 Da) at *m*/*z* 315. Flavanonol subclass as (+−)-taxifolin was also identified at *m*/*z* 303.05.

The isoflavonoid deprotonated ion at *m*/*z* 415.15 was identified for daidzein-8-*C*-glucoside with neutral loss of glucose (162 Da) at *m*/*z* 253 [M-H-glc]-. Formononetin was also detected at *m*/*z* 267.08 as 4’-*O*-methyl isoflavones.

Two biflavonoids detected in EVME were procyanidin B1 (13) and B2 (23) at *m*/*z* 577.13 and 579.14 in negative mode. 

#### 3.1.3. Identification of Coumarins and Other Derivatives

Two coumarins were detected as esculin and daphnetin [M-H]^−^ at *m*/*z* 339.12 and 177.01, respectively. Esculin is a coumarin glycoside derivative, with MS/MS fragments at *m*/*z* 295 due to neutral loss of CO_2_ (44 Da), 175 [M-2H-glc]-, and 149 [C_5_H_10_O_5_]-H-. Daphnetin is 7,8-dihydroxycoumarins identified with MS/MS fragments at *m*/*z* 117.09, 133.03, 149.02, 163.01 and 177.01.

Two stilbenes’ derivatives were detected—*E*-3,4,5’-trihydroxy-3’-glucopyranosyl stilbene, showing [M-H]^−^ at *m*/*z* 405.01, with a loss of 162 Da of glucose at *m*/*z* 243.02, and *E*-4,5’-dihydroxy-3-methoxy-3’-glucopyranosylstilbene, which showed [M-H]^−^ at *m*/*z* 419.07.

Only one catechin was detected in EVME at *m*/*z* 289.07. Another detected compound in EVME was hinokitiol. It is a tropolone monoterpenoid derivative detected at *m*/*z* 163.07.

### 3.2. In Vitro Antifungal Activity

#### 3.2.1. Antifungal Susceptibility Testing and Determination of MIC

A total of 12 *C. glabrata* clinical isolates were identified with microscopical examination and MALDI–TOF. In addition, EVME exhibited antifungal activity against the tested isolates using the agar well diffusion method. The MIC values of EVME were determined using the broth microdilution method, and they ranged from 32 to 256 µg/mL, as shown in Table S2.

#### 3.2.2. Time–Kill Study

**Figure 2:**
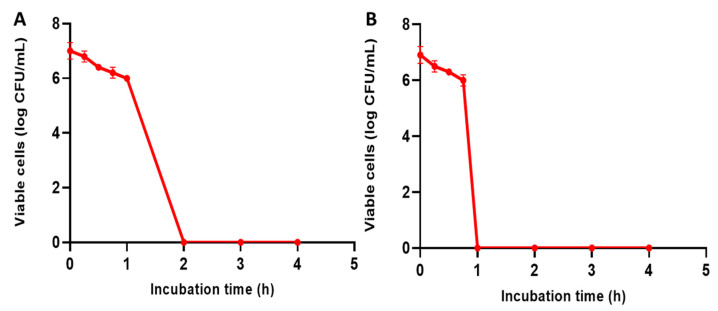
Time–kill curve of representative *C. glabrata* isolates showing a decrease in the count of CFU/mL by three log units after treatment with EVME with concentrations of (**A**) 1× minimum inhibitory concentration (MIC) for two hours and (**B**) 4× MIC for one hour.

We observed that the count of CFU/mL of *C. glabrata* isolates declined by more than three log units when the isolates were incubated with EVME at concentrations of 1× MIC for two hours and 4× MIC for one hour. The isolates that exhibited this decline were 41.67% and 50%, respectively. A demonstrative illustration of this decline in the number of CFU/mL is revealed in Figure 2.

#### 3.2.3. Nucleotide Leakage

**Figure 3:**
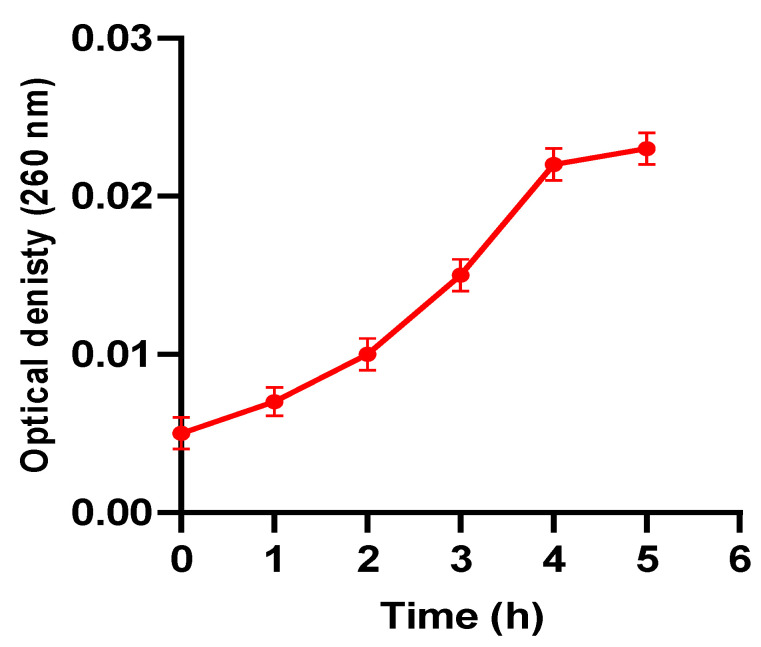
A representative example of the significant decrease (*p* < 0.05) in the membrane integrity by EVME by increasing the leakage of nucleic acids.

The fungal cell membrane integrity was evaluated after treatment with EVME (using 0.5 MIC values). This was performed by detecting the discharge of the nucleic acid materials, which absorb at 260 nm, from the fungal cells. EVME was found to significantly decrease (*p* < 0.05) the membrane integrity of 41.67% of *C. glabrata* isolates. A representative illustration is revealed in Figure 3.

#### 3.2.4. Sorbitol Protection Assay

**Figure 4:**
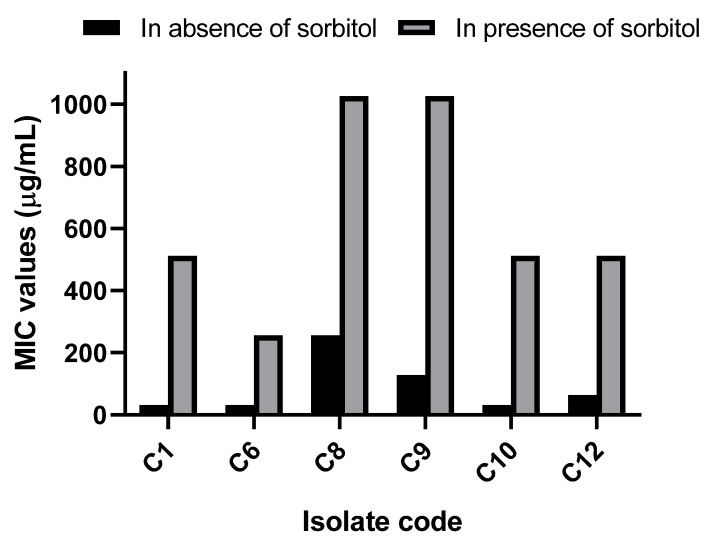
Minimum inhibitory concentration (MIC) values of EVME of six *C. glabrata* isolates showed a substantial increase (*p* < 0.05) in the presence of sorbitol.

Sorbitol protection assay was carried out to further study the impact of EVME on the fungal cell membrane. Interestingly, we noticed a substantial increase (*p* < 0.05) in the MIC values of EVME in the presence of sorbitol in 50% of the isolates, as shown in Figure 4. 

#### 3.2.5. Phenotypic and Genotypic Detection of the Impact of EVME on Efflux Pumps

The effects of EVME on the efflux activity of *C. glabrata* isolates were tested using the acridine orange test. It was observed that EVME resulted in a decrease in the percentage of the *C. glabrata* isolates having strong and moderate levels of efflux activity from 50% to 16.67%, as shown in Table 2.

**Figure 5:**
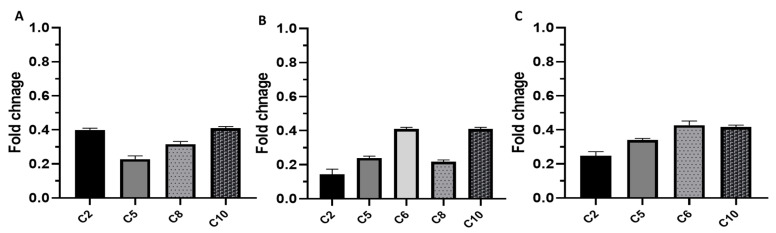
Downregulation of the gene expression of (**A**) *CDR1*, (**B**) *CDR2*, and (**C**) *ERG11* after treatment with EVME in *C. glabrata* isolates C2, C5, C6, C8, C10.

The impact of EVME on the efflux activity of *C. glabrata* isolates was studied in depth using qRT-PCR to elucidate the effect of EVME on the expression of the genes encoding efflux pumps. Interestingly, EVME was found to produce downregulation of the expression of *CDR1*, *CDR2*, and *ERG11* genes in the tested isolates with percentages of 33.33%, 41.67%, and 33.33%, respectively, as shown in Figure 5.

### 3.3. In Vivo Antifungal Activity

#### 3.3.1. Fungal Burden in the Kidney Tissues and Survival Rate

**Figure 6:**
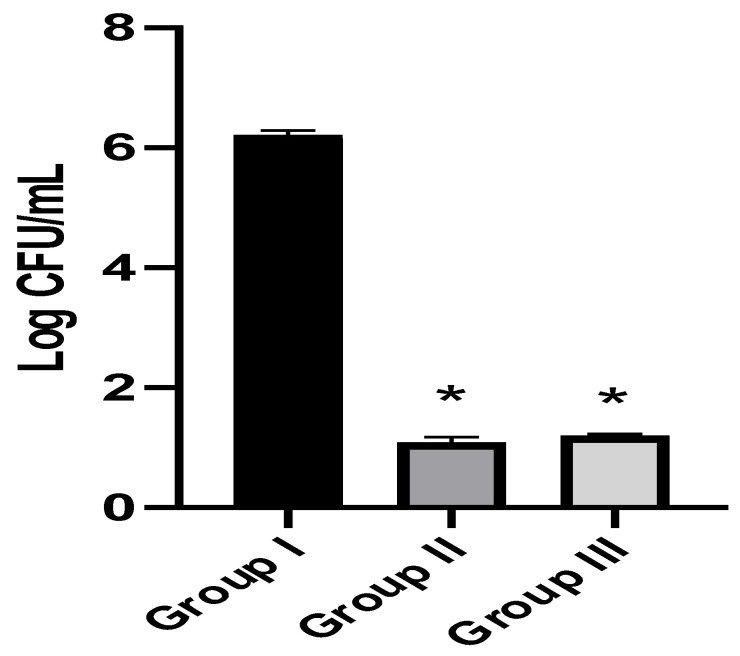
The number of colony-forming units per gram (CFU/g) of fungal cells in kidneys of the three groups. The symbol (*) represents a substantial decrease (*p* < 0.05). There was a non-significant difference between group II and group III.

We counted the number of colony-forming units per gram (CFU/g) of the fungal cells in the kidney tissues in the tested experimental groups. EVME was found to reduce significantly (*p* < 0.05) the number of colony-forming units per gram (CFU/g) of fungal cells in kidneys, as shown in Figure 6.

**Figure 7:**
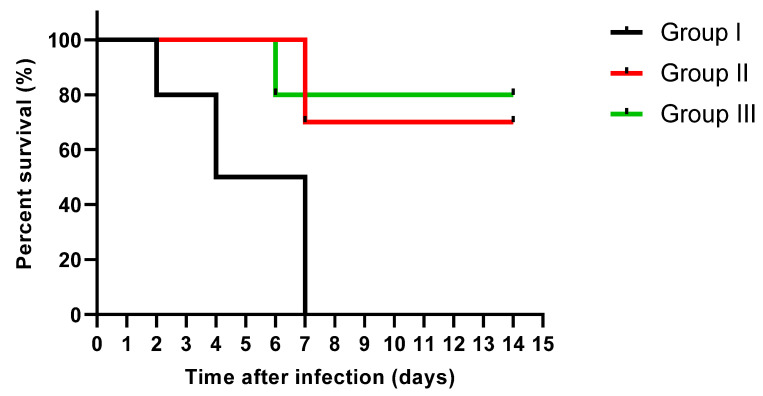
Survival curve of rats of the different groups via Kaplan–Meier survival analysis.

Moreover, the survival curve was constructed, as shown in Figure 7. Two rats in group I died after two days, three rats died after four days, and the rest after one week. Only three rats died after one week in group II, and the rest remained alive till the 14th day. Only two rats died after six days in group III, and the other rats remained alive till the 14th day.

#### 3.3.2. Kidney Function Test

Both blood urea and serum creatinine levels were determined in the three tested groups. As shown in Table 3, there were substantial increases (*p* < 0.05) in blood urea and serum creatinine levels in group I, compared with the other two groups.

#### 3.3.3. Histological Assessment

**Figure 8:**
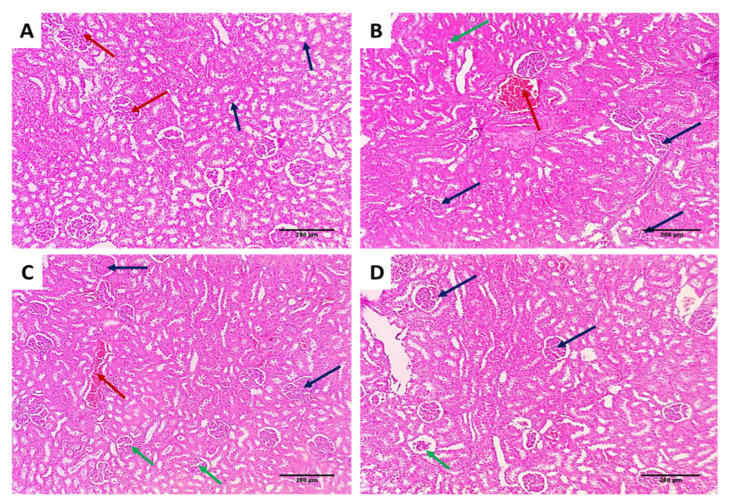
H&E-stained kidney sections of (**A**) normal kidney tissues (negative control) showing average-sized glomeruli (red arrows) surrounded by average-sized tubules lined with columnar cells (blue arrows) (×100); (**B**) group I showing vascular congestion (red arrow) surrounded by atrophic glomeruli (blue arrows) and focal degenerated tubules (green arrow) (×100); (**C**) group II showing focal vascular congestion (red arrow) surrounded by normal-sized glomeruli (blue arrows) and some atrophic glomeruli (green arrows) surrounded by average-sized tubules (×100); (**D**) group III shows average-sized glomeruli (blue arrows) and one atrophic glomerulus (green arrow) surrounded by average-sized tubules (×100).

Kidney tissues from the different groups were examined for assessment of the effect of EVME on the kidney tissues of the rats infected with *C. glabrata,* as shown in Figure 8.

**Figure 9:**
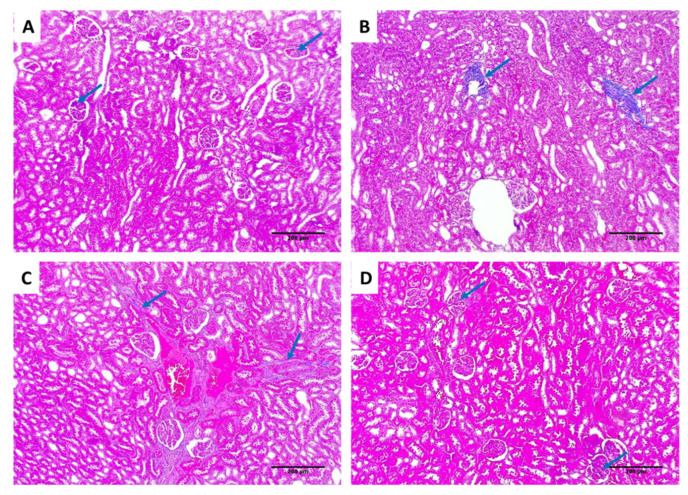
Masson’s trichrome staining of the kidney sections of (**A**) normal kidney tissues (negative control) showing slight amounts of blue-stained collagen fibers in glomeruli (blue arrows) (×100); (**B**) group I showing a marked increase in the abnormal collagen deposition in the walls of tubules (blue arrows) (×100); (**C**) group II showing vascular congestion with focal blue-stained collagen fibers deposition around the vessels (blue arrows), no collagen deposition in the tubules (×100); (**D**) group III showing a marked reduction in the abnormal collagen fibers deposition and slight amounts in the glomeruli (blue arrows), with no collagen deposition in the tubules or the blood vessels (×100).

Collagen staining of the kidney tissues of the different groups using Masson’s trichrome stain is shown in Figure 9.

#### 3.3.4. Immunohistochemical Studies

**Figure 10:**
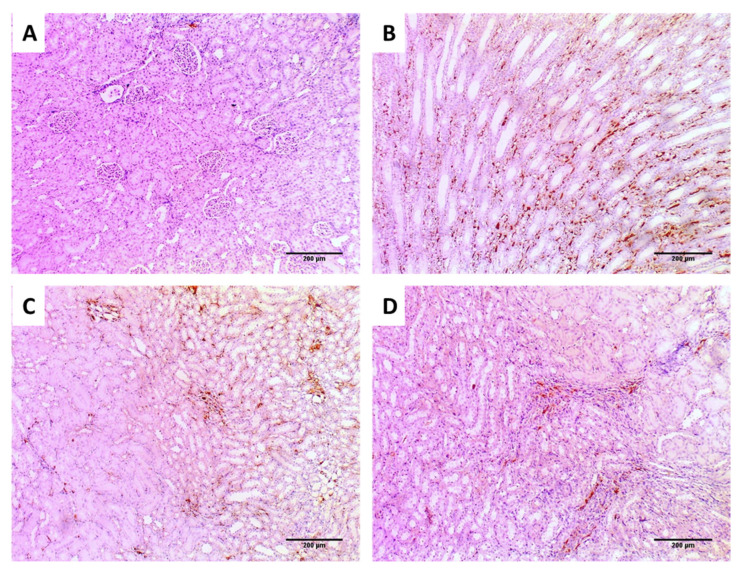
Tumor necrosis factor-alpha (TNF-α) immunohistochemical staining of the kidney sections of (**A**) normal kidney tissues (negative control) showing negative TNF-α staining, with score (0) (×100); (**B**) group I showing strong TNF-α staining with score (3) (×100); (**C**) group II showing moderate TNF-α staining with score (2) (×100); (**D**) group III showing weak TNF-α staining with score (1) (×100).

We accomplished immunostaining of the kidney tissues of the different groups using TNF-α, as shown in Figure 10.

## 4. Discussion

*Candida* spp. can cause opportunistic infections called candidiasis, which can occur in different parts of the body. When *Candida* spp. occur in the bloodstream, it is called candidemia, which has a very high mortality rate [34]. *C. glabrata* can cause both superficial and systemic infections, especially in immunocompromised patients [34]. Thus, in our study, we investigated the antifungal activity of EVME against *C. glabrata* clinical isolates in vitro and in vivo. Several researchers have reported the activity of certain plant extracts against candidiasis caused by different *Candida* spp. [35,36]. For instance, Bonifácio et al. [36] elucidated the effectiveness of plant extracts belonging to *Astronium* sp. against *C. albicans*. Additionally, Oliveira et al. elucidated the effectiveness of Myracrodruon urundeuva plant extract against *Candida* sp. [20]. However, most of the conducted research focused on *C. albicans*, and little attention has been given to *C. glabrata.* Nevertheless, infections caused by *C. glabrata* have increased in recent decades due to its growing resistance to azoles [1].

Medicinal plants have several secondary metabolites and bioactive compounds with various biological activities [37,38]. However, many of them remain unexplored yet. The antifungal effect of plant extracts is an important biological activity that should be investigated owing to the insufficiency of the currently present antifungal drugs. Many fungi, especially *Candida* spp., are acquiring resistance to antifungals. Additionally, many antifungals have various side effects, which restrict their use [39]. 

LC–ESI–MS/MS of EVME identified 51 molecules belonging to several phytochemical subclasses. This investigation revealed that EVME possesses antimicrobial and anti-inflammatory activities, which are likely due to the presence of several active constituents, such as phenolics, flavonoids, biflavonoids, and their glycoside derivatives, terpenoid, and coumarin derivatives [40,41,42,43]. According to peak height and area measurements, D-(−)-quinic acid is the principal acid present in EVME. Naringenin is the most abundant metabolite, followed by hesperetin and apigenin, while apigenin-7-*O*-glucoside is the most abundant flavonoid glycoside, followed by luteolin-7-*O*-glucoside. 

The antimicrobial and anti-inflammatory of EVME were consistent with prior research on the anti-inflammatory and antimicrobial properties of certain flavonoids or flavonoid glycosides, such as apigenin, kaempferol, and luteolin derivatives [14,44,45], as well as naringenin [37], daidzein [46], biflavonoid derivatives [19,37,47,48], and coumarin derivatives [49,50].

Herein, EVME exhibited antifungal activity against *C. glabrata* clinical isolates with MIC values that ranged from 32 to 256 µg/mL. Therefore, we carried out time–kill studies to elucidate the pattern of growth and killing rate of the isolates by EVME [51,52]. This was performed to illustrate the dynamic interaction between EVME and *C. glabrata* isolates. In addition, the nucleotide leakage and sorbitol protection assays were carried out to reveal if EVME has an impact on the fungal cell membrane and cell wall.

There was a significant decrease (*p* < 0.05) in the membrane integrity caused by EVME in 41.67% of *C. glabrata* isolates. This finding suggests that EVME could have a disruptive impact on the fungal cell membrane. The cell membrane of fungi is a target for certain antifungals. Thus, it was studied by many researchers [53,54]. In addition, the MIC values of EVME exhibited a significant rise (*p* < 0.05) in the presence of sorbitol in 50% of the isolates. Sorbitol has an osmoprotective property meaning that it can help cells with impaired cell walls to grow [18,55].

Efflux pumps are a common cause of fungi resistance to antifungals, and they are encoded by different genes [3]. Therefore, the impact of EVME on the efflux pump activity was studied, both phenotypically using acridine orange and genotypically [56]. qRT-PCR is an efficient, cost-effective, and relatively simple technique that can be used to quantify the expression of the genes [57,58]. Therefore, this technique was used to study the genotypic impact of EVME on the efflux genes. EVME resulted in a downregulation of *CDR1*, *CDR2*, and *ERG11* genes in the tested isolates, with percentages of 33.33%, 41.67%, and 33.33%, respectively. Thus, EVME could be a promising source for efflux pump inhibitor compounds that can lead to decreasing the resistance to the antifungal drugs.

Regarding the in vivo model, we selected a systemic infection model for investigating the effectiveness of EVME against *C. glabrata* fungal isolates to mimic the actual real infection. Interestingly, EVME significantly reduced (*p* < 0.05) the number of CFU/g of fungal cells in kidney tissues and increased the infected rats’ survival rates. In addition, the kidney tissues were studied using histopathological and immunohistochemical investigations. We found that EVME resulted in average-sized glomeruli and tubules in comparison with group I. Moreover, it resulted in decreasing the proinflammatory cytokine TNF-α, which has a role in the inflammatory process [11,59]. Regarding the collagen staining using Masson’s trichrome stain, it revealed a marked reduction in the abnormal collagen fibers deposition and protection of the kidney from pathological renal fibrosis.

## 5. Conclusions

LC–ESI–MS/MS contributed to the authentication of the investigated plant and identified 51 active constituents, including flavonoids, flavonoid glycosides, phenolic, carboxylic, and fatty acids, that were probably accountable for the *E. villosus*-exhibited effects. In addition, EVME revealed a promising antifungal activity against *C. glabrata* fungal isolates in vitro by disturbing the membrane integrity and cell wall and inhibiting the efflux pump activity by affecting its gene expression. Moreover, it exhibited antifungal activity in vivo by decreasing the congestion and production of average-sized glomeruli and tubules. It resulted in an improvement of the kidney function tests, with decreasing TNF-α and collagen fibers deposition.

As a final point, the current study highlighted *E. villosus* as a rich source of antifungal compounds. Therefore, more studies should be performed on EVME to isolate the pure active ingredients and to determine the compounds responsible for the activity. Additionally, preclinical and clinical studies should be performed to elucidate its effectiveness as a new antifungal agent. 

## Figures and Tables

**Table 1:** Phytochemical profiling of EVME via LC–MS/MS (negative mode) analysis.

No.	Error	Metabolite Name	Rt Min	Molecular Formula	Identification		Fragments or MS^2^ Ions *m*/*z*	Precursor *m*/*z* [M–H]^−^	Theoretical Mass	Ref.
			**Phenolic or Organic Acids Derivatives**							
1	−4.7	D-Malic acid	1.098	C_4_H_6_O_5_	Beta hydroxy acids and derivatives		59.01, 71.01, 115.00 133.01	133.0150	134.0230	[22]
2	−2.1	Shikimic acid	1.111	C_7_H_10_O_5_	Shikimic acids and derivatives		73.02, 137.04, 173.04	173.0460	174.0540	[23]
3	−0.1	Maleic acid	1.112	C_4_H_4_O_4_	Dicarboxylic acids and derivatives		71.01, 115.01	115.0032	116.0112	
4	−0.2	D-Quinic acid	1.136	C_7_H_12_O_6_	Quinic acids and derivatives		85.02, 145.04, 173.04, 191.05	191.0562	192.0642	
5	−0.9	3,4-Dihydroxy benzoic acid	1.257	C_7_H_6_O_4_	Hydroxybenzoic acid derivatives		109.03, 153.01	153.0187	154.0267	[24]
6	3.1	Caffeic acid	1.344	C_9_H_8_O_4_	Hydroxycinnamic acids		59.02, 89.02, 135.04, 179.05	179.0545	180.0625	
7	4.4	3-(4-Hydroxy-3-methoxy-phenyl) prop-2-enoic acid	2.076	C_10_H_10_O_4_	Hydroxycinnamic acids		134.03, 193.05	193.0524	194.0604	
8	0.8	Salicylic acid	4.442	C_7_H_6_O_3_	Salicylic acid		91.02, 108.01, 136.01, 137.02	137.0238	138.0318	[25]
9	0.8	(+/−)-cis,trans-abscisic acid	5.286	C_15_H_20_O_4_	Abscisic acids and derivatives		151.07, 219.13, 263.11	263.1278	264.1358	[25]
10	−0.7	5-Methoxy salicylic acid	6.857	C_8_H_8_O_4_	M-Methoxybenzoic acids and derivatives		108.02, 152.01, 167.03	167.0352	168.0432	
11	6.0	Rosmarinic acid	7.136	C_18_H_16_O_8_	Coumaric acids and derivatives		181.04, 329.09, 359.11	359.1105	360.1185	[26]
12	−3.1	Isocitrate	27.721	C_6_H_8_O_7_	Tricarboxylic acids and derivatives		150.96, 190.95	191.1050	192.1130	
			**Flavonoids and related metabolites**							
13	−3.7	Baicalein-7-*O*-glucuronide	1.293	C_21_H_18_O_11_	445.07	Flavonoid-7-*O*-glucuronides	102.95, 191.05, 269.02 377.07, 445.14	445.1807	446.1887	[27]
14	−2.1	Isorhamnetin-3-*O*-rutinoside	4.930	C_28_H_32_O_16_	623.12	Flavonoid-3-*O*-glycosides	285.03, 470.87, 579.00, 623.12	623.1269	624.1349	[28]
15	−3.1	Procyanidin B2	5.006	C_30_H_26_O_12_	577.13	Biflavonoids and polyflavonoids	407.07, 425.08, 577.13	577.1369	578.1449	[28]
16	5.0	Kaempferol-3-*O*-alpha-L-rhamnoside	5.223	C_21_H_20_O_10_	431.18	Flavonoid-3-*O*-glycosides	151.04, 179.05, 362.88, 431.19	431.1890	432.1970	
17	−3.6	Daidzein-8-C-glucoside	5.452	C_21_H_20_O_9_	415.15	Isoflavonoid C-glycosides	210.92, 286.92, 415.16	415.1572	416.1652	[29, 30]
18	−0.1	Naringenin-7-*O*-glucoside	6.170	C_21_H_22_O_10_	433.20	Flavonoid-7-*O*-glycosides	271.06, 387.18, 433.10	433.2068	434.2148	
19	−2.2	(+−)-Taxifolin	6.492	C_15_H_12_O_7_	303.05	Flavanonols	125.02, 179.00, 1990.95, 285.04, 303.04	303.0520	304.0600	
20	2.5	Hyperoside	6.542	C_21_H_20_O_12_	463.08	Flavonoid-3-*O*-glycosides	218.94, 286.94. 300.02, 326.92, 394.90, 463.08	463.0858	464.0938	
21	0.5	Apigenin 8-*C*-glucoside	6.542	C_21_H_20_O_10_	431.09	Flavonoid 8-*C*-glycosides	269.04,311.05, 362.89, 431.20	431.0956	432.1036	
22	−0.1	Formononetin	6.734	C_16_H_12_O_4_	267.08	4’-*O*-methylisoflavones	133.02, 193.04, 252.06, 267.08	267.0875	268.0955	
23	0.9	Procyanidin B1	6.785	C_30_H_26_O_12_	577.14	Biflavonoids and polyflavonoids	245.04, 425.08, 577.12	577.1337	578.1417	[31]
24	0.6	Luteolin-7-*O*-glucoside	6.913	C_21_H_20_O_11_	447.09	Flavonoid-7-*O*-glycosides	285.03, 327.04, 447.09	447.0923	448.1003	
25	8.9	Isookanin-7-glucoside	6.926	C_21_H_22_O_11_	449.09	Flavonoid-7-*O*-glycosides	151.00, 287.04, 449.09	449.0952	450.1032	
26	0.4	Isorhamnetin-3-*O*-glucoside	7.417	C_22_H_22_O_12_	477.10	Flavonoid-3-*O*-glycosides	285.03, 299.01, 315.04, 477.09	477.1033	478.1113	
27	−0.1	Kaempferol-7-neohesperidoside	7.539	C_27_H_30_O_15_	593.14	Flavonoid-7-*O*-glycosides	269.04, 593.13,	593.1483	594.1563	
28	−0.9	Apigenin-7-*O*-glucoside	7.700	C_21_H_20_O_10_	431.09	Flavonoid-7-*O*-glycosides	268.03, 311.06, 431.09	431.0988	432.1068	
29	10.9	Kaempferol-3-Glucuronide	7.893	C_21_H_18_O_12_	461.10	Flavonoid-3-*O*-glucuronides	256.92, 299.09, 392.89, 461.09	461.1031	462.1111	
30	−3.7	3’ 4’ 5 7-Tetrahydroxy flavanone	8.678	C_13_H_12_O_6_	287.05	Flavanones	135.04, 151.00, 218.94, 287.06	287.0984	288.1064	
31	−0.2	Kaempferol-3-*O*-alpha-L-arabinoside	8.751	C_20_H_18_O_10_	417.11	Flavonoid-3-*O*-glucuronides	179.05, 362.88, 431.19	417.1194	418.1274	
32	−0.1	Naringenin	9.744	C_15_H_12_O_5_	271.09	Flavanones	228.07, 255.05, 271.09	271.0985	272.1065	[32]
33	−2.6	3’-Methoxy-4’,5,7-trihydroxy flavonol	10.368	C_16_H_12_O_7_	315.05	Flavonols	257.04, 300.02, 315.04	315.0528	316.0608	
34	−3.0	Apigenin	10.428	C_15_H_10_O_5_	269.04	Flavones	117.03, 151.00, 181.06, 225.05, 269.04	269.0467	270.0547	[32]
35	0.8	Luteolin	11.175	C_15_H_10_O_6_	285.07	Flavones	151.00, 270.06, 263.11	285.0763	286.0843	[32]
36	0.7	Rhoifolin	11.421	C_27_H_30_O_14_	577.13	Flavonoid-7-*O*-glycosides	291.19, 464.94, 577.13	577.1346	578.1426	
37	−4.0	3 5 7-Trihydroxy-4’-methoxyflavone	12.886	C_16_H_12_O_6_	299.09	Flavonols	119.04, 179.03, 193.05, 299.09	299.0558	300.0638	
38	−0.1	4’,5,7-Trihydroxy flavonol	13.890	C_15_H_10_O_6_	285.11	Flavonols	119.04, 149.99, 165.02, 285.07	285.1115	286.1195	
39	−0.4	Hesperetin	14.598	C_16_H_14_O_6_	301.10	4’-*O*-methylated flavonoids	138.03, 151.03, 286.08, 301.10	301.0692	302.0772	[33]
40	−0.2	Luteolin-3’, 7-di-*O*-glucoside	17.799	C_27_H_30_O_16_	609.13	Flavonoid-7-*O*-glycosides	564.90, 594.11, 609.13	609.1440	610.1520	
			**Fatty acids**							
41	−0.8	3-Hydroxy-3-Methyl glutaric acid	1.159	C_6_H_10_O_5_	161.04	Hydroxy fatty acids	57.03, 99.04, 161.04	161.0452	162.0532	
42	−2.7	2-Isopropyl malic acid	1.331	C_7_H_12_O_5_	175.05	Hydroxy fatty acids	101.02, 113.05, 175.06	175.0599	176.0679	
43	−0.5	Citraconic acid	1.394	C_5_H_6_O_4_	129.01	Methyl-branched fatty acids	84.99, 129.01	129.0189	130.0269	
44	1.5	Citramalate	1.407	C_5_H_8_O_5_	147.06	Hydroxy fatty acids	72.98, 87.00, 129.01, 147.06	147.0647	148.0727	
45	−4.0	Gamma-Linolenic acid	18.900	C_18_H_30_O_2_	277.14	Linoleic acids and derivatives	141.09, 233.15, 277.13	277.1465	278.1545	
			**Others**							
46	−0.3	E-3,4,5’-Trihydroxy-3’-glucopyranosyl stilbene	1.123	C_20_H_22_O_9_	405.01	Stilbene glycosides	190.96, 191.05, 243.02, 369.03, 405.09	405.1208	406.1288	
47	−0.6	Esculin	1.172	C_15_H_16_O_9_	339.12	Coumarin glycosides	149.01, 175.02, 202.90, 295.10, 339.11	339.1296	340.1376	
48	0.2	Catechin	5.452	C_15_H_14_O_6_	289.07	Catechins	109.02, 125.02, 179.03, 203.03, 245.07, 289.06	289.0711	290.0791	[31]
49	3.0	Hinokitiol	6.815	C_10_H_12_O_2_	163.07	Tropolones	119.08, 135.08, 163.07	163.0751	164.0831	
50	−0.4	Daphnetin	7.295	C_9_H_6_O_4_	177.01	7,8-Dihydroxycoumarins	117.09, 133.03, 149.02, 163.01, 177.01	177.018	178.0260	
51	1.0	*E*-4,5’-Dihydroxy-3-methoxy-3’-glucopyranosyl stilbene	9.547	C_21_H_24_O_9_	419.07	Stilbene glycosides	257.02, 343.01, 363.12, 419.06	419.0748	420.0828	

**Table 2:** Impact of EVME on efflux pump activity of *C. glabrata* isolates.

Efflux Pump Activity	No. of Isolates	
	Before Treatment	After Treatment
Strong	3	1
Moderate	3	1
Weak	4	6
None	2	4

**Table 3:** Blood urea and serum creatinine levels in the tested groups.

Measured Parameter	Group I	Group II	Group III
Blood urea level (mg/dL)	69 ± 1.40 *	47.6 ± 1.10	48 ± 0.90
Serum creatinine level (mg/dL)	0.95 ± 0.06 *	0.7 ± 0.02	0.69 ± 0.06

The symbol * represents a significant difference from the other two groups (*p* < 0.05).

## Data Availability

All data are contained within the article and Supplementary Materials.

## Supplementary Materials

The following are available online at https://www.mdpi.com/article/10.3390/jof8050521/s1. Table S1: Sequences of the utilized primers, Table S2: MIC values of EVME against the tested *C. glabrata* isolates, Figure S1: MS2 spectral fragmentation of all identified metabolites.
