# Supplementary material for: Encephalartos villosus Lem. Displays a Strong In Vivo and In Vitro Antifungal Potential against Candida glabrata Clinical Isolates
Source: J Fungi (Basel). 2022 May 18;8(5):521. doi: 10.3390/jof8050521 (PMC9146621; doi:10.3390/jof8050521)
Supplement: Supplementary file 1 [file jof-08-00521-s001.zip › jof-1713708-supplementary.pdf]

Table S1. Sequences of the utilized primers.

Genes	Sequence	
CDR1	F<br>R	5′-TAGCACATCAACTACACGAACGT-3′<br>5′-AGAGTGAACATTAAGGATGCCATG-3′
CDR2	F<br>R	5′-GTGCTTTATGAAGGCTACCAGATT-3′<br>5′-TCTTAGGACAGAAGTAACCCATCT-3′
ERG11	F<br>R	5′-ATTGGTGTCTTGATGGGTGGTC-3′<br>5′-TCTTCTTGGACATCTGGTCTTTCA-3′
URA3 (reference gene)	F<br>R	5′-GAAAACCAATCTTTGTGCTTCTCT-3′<br>5′-CATGAGTCTTAAGCAAGCAAATGT-3′

Table S2. MIC Values of EVME against the tested *C. glabrata* isolates.

Isolate code	MIC value (μg/mL)	Isolate code	MIC value (μg/mL)
C1	32	C7	128
C2	32	C8	256
C3	256	C9	128
C4	64	C10	32
C5	128	C11	128
C6	32	C12	64

Figure S1. MS^2 spectral fragmentation of all identified metabolites.

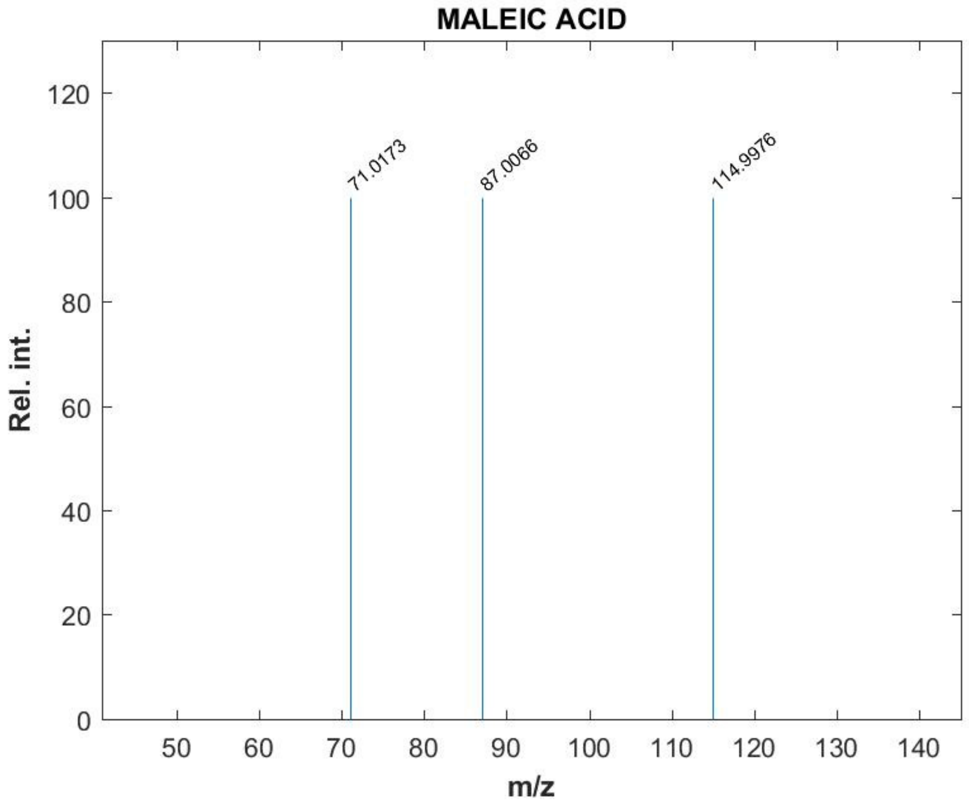

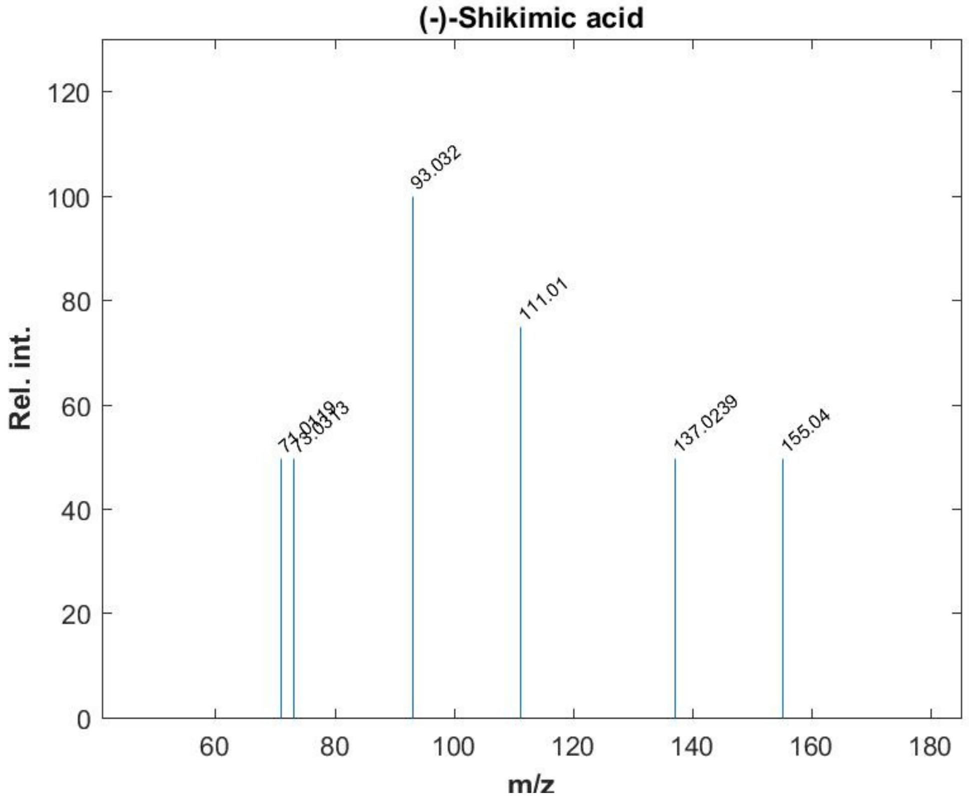
(-)-Shikimic acid
Rel. int.
m/z
93.032
111.01
71.0119
73.0313
137.0239
155.04

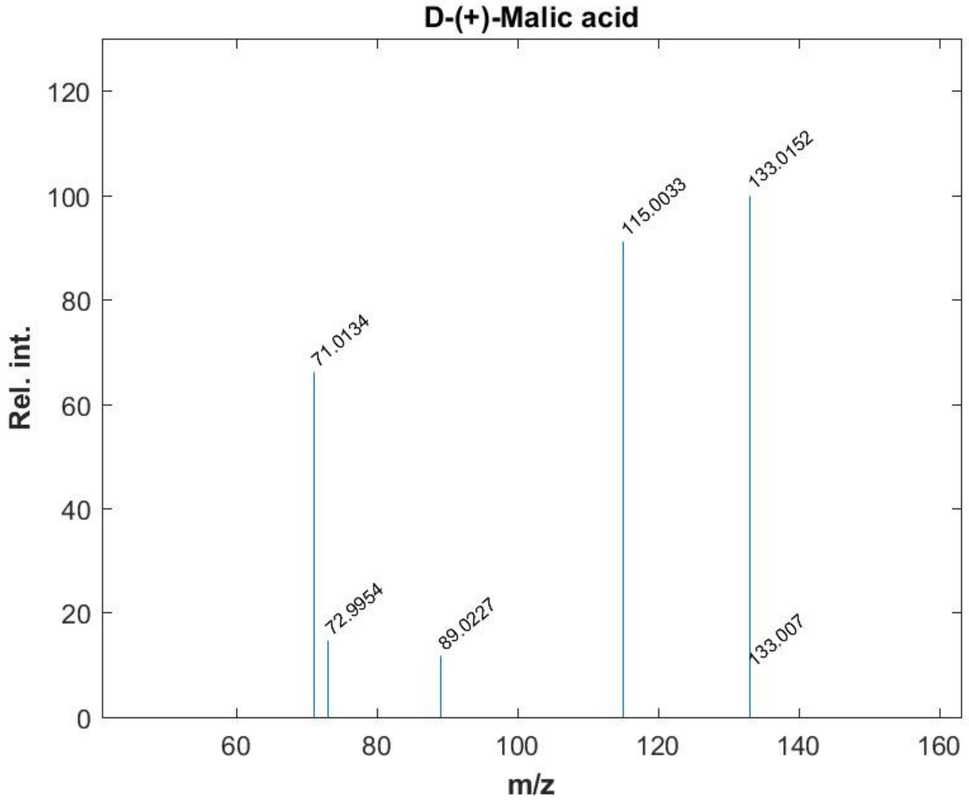
D-(+)-Malic acid
Rel. int.
m/z
133.0152
115.0033
71.0134
72.9954
89.0227
133.007

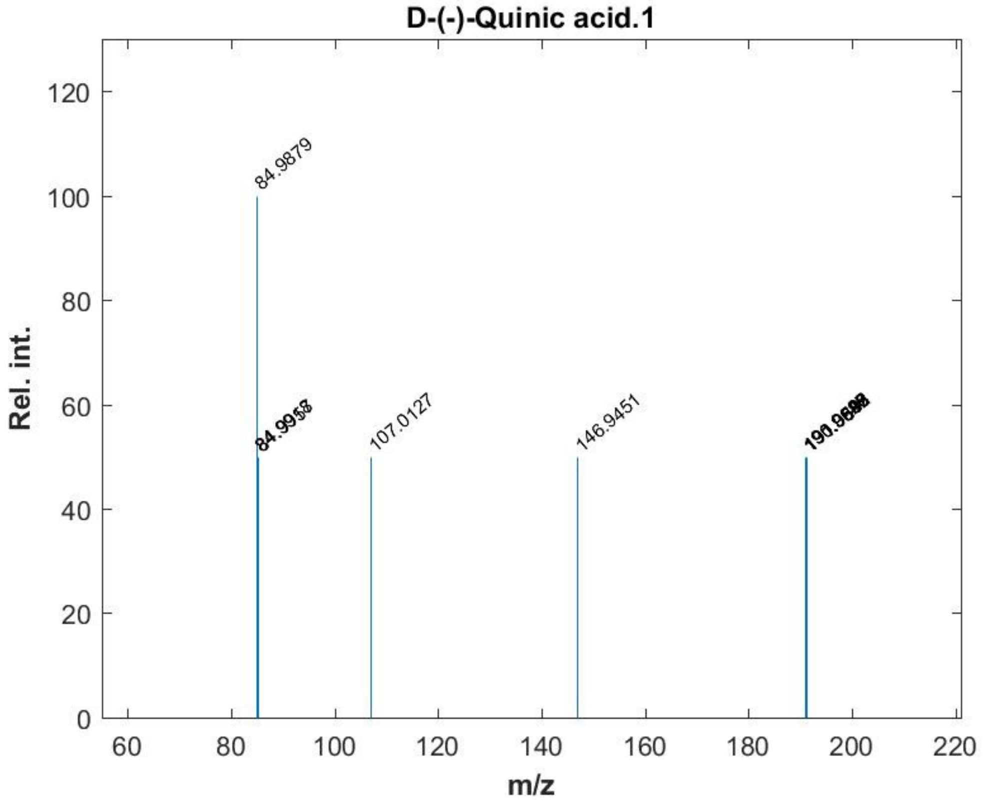
D-(-)-Quinic acid.1
Rel. int.
m/z
84.9879
107.0127
146.9451

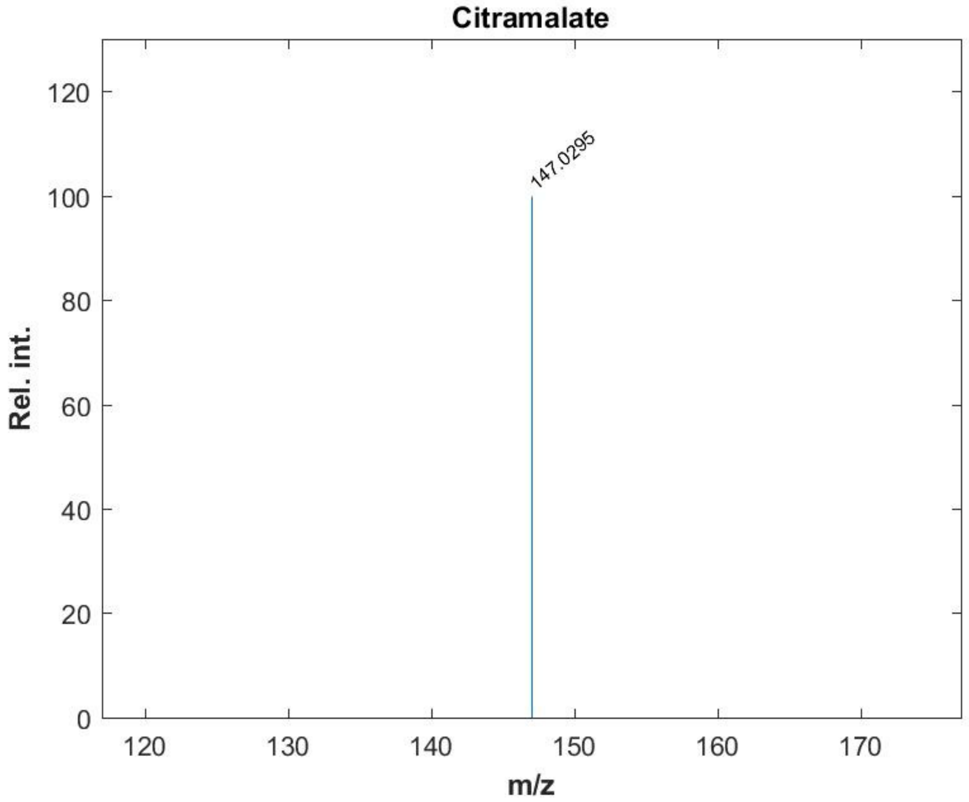
Citramalate
Rel. int.
m/z
147.0295
0
20
40
60
80
100
120
120
130
140
150
160
170

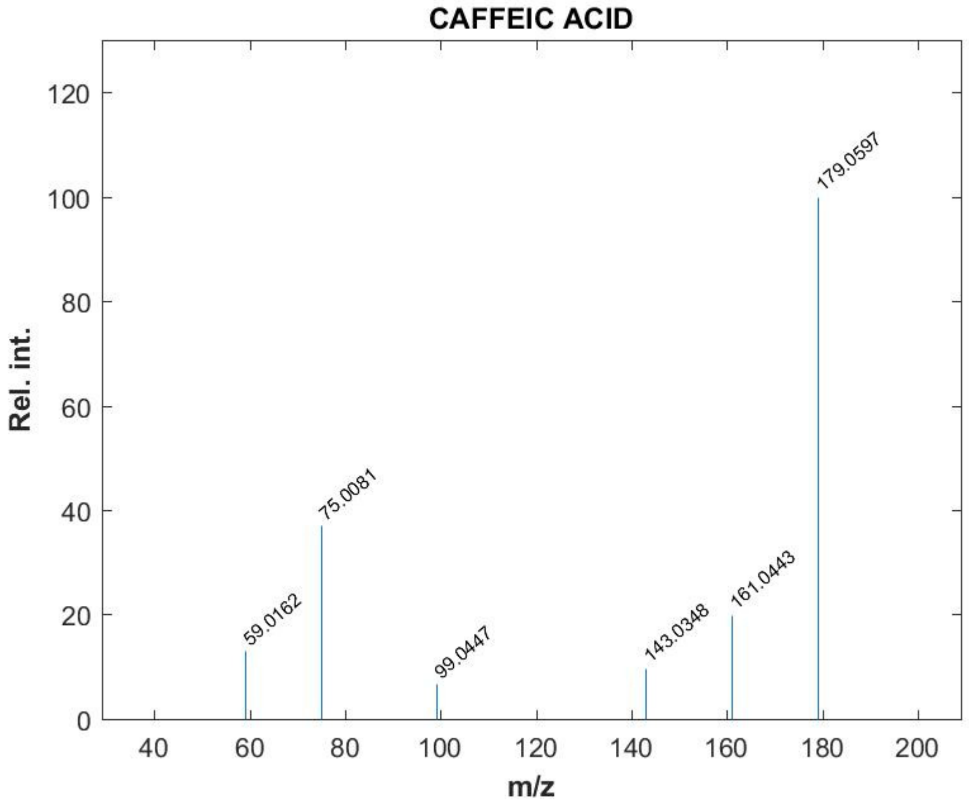
CAFFEIC ACID
Rel. int.
m/z
179.0597
75.0081
59.0162
99.0447
143.0348
161.0443
0
20
40
60
80
100
120
40
60
80
100
120
140
160
180
200

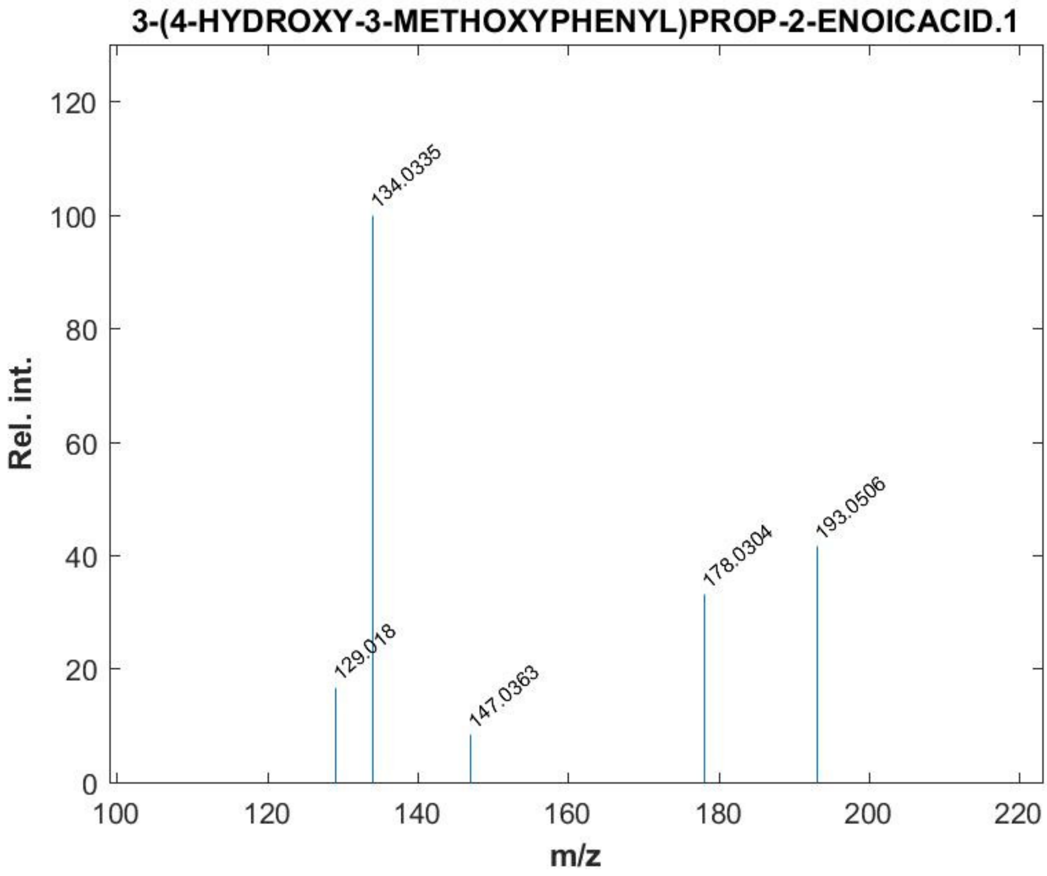
3-(4-HYDROXY-3-METHOXYPHENYL)PROP-2-ENOICACID.1
Rel. int.
m/z
134.0335
129.018
147.0363
178.0304
193.0506

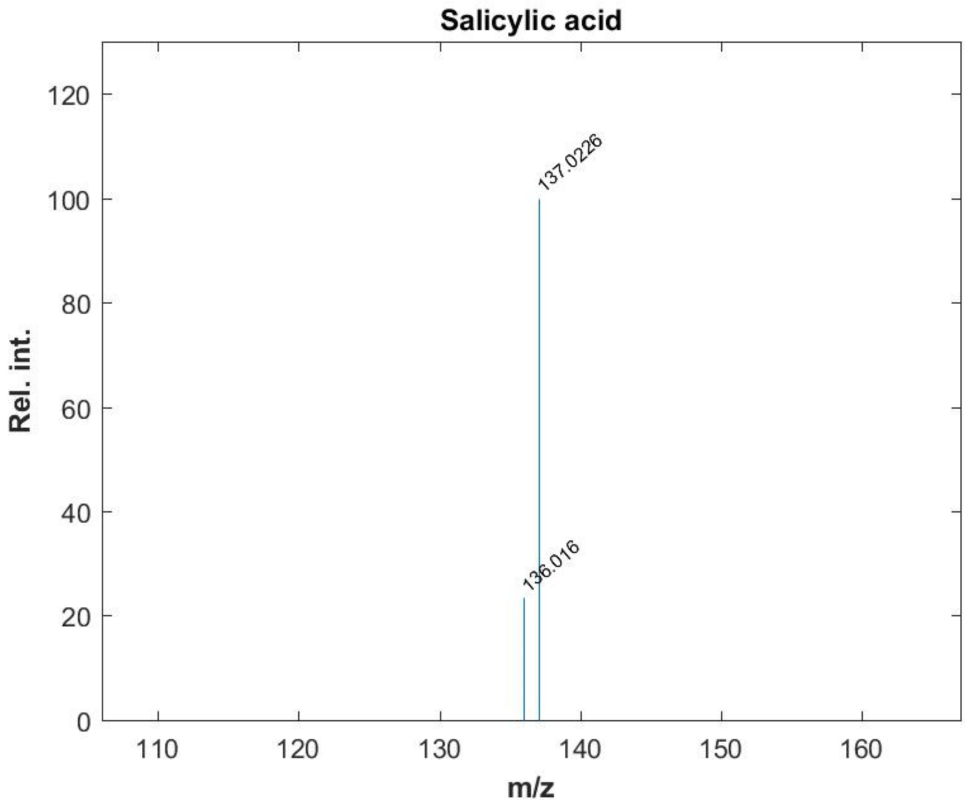
Salicylic acid
Rel. int.
m/z
137.0226
136.016

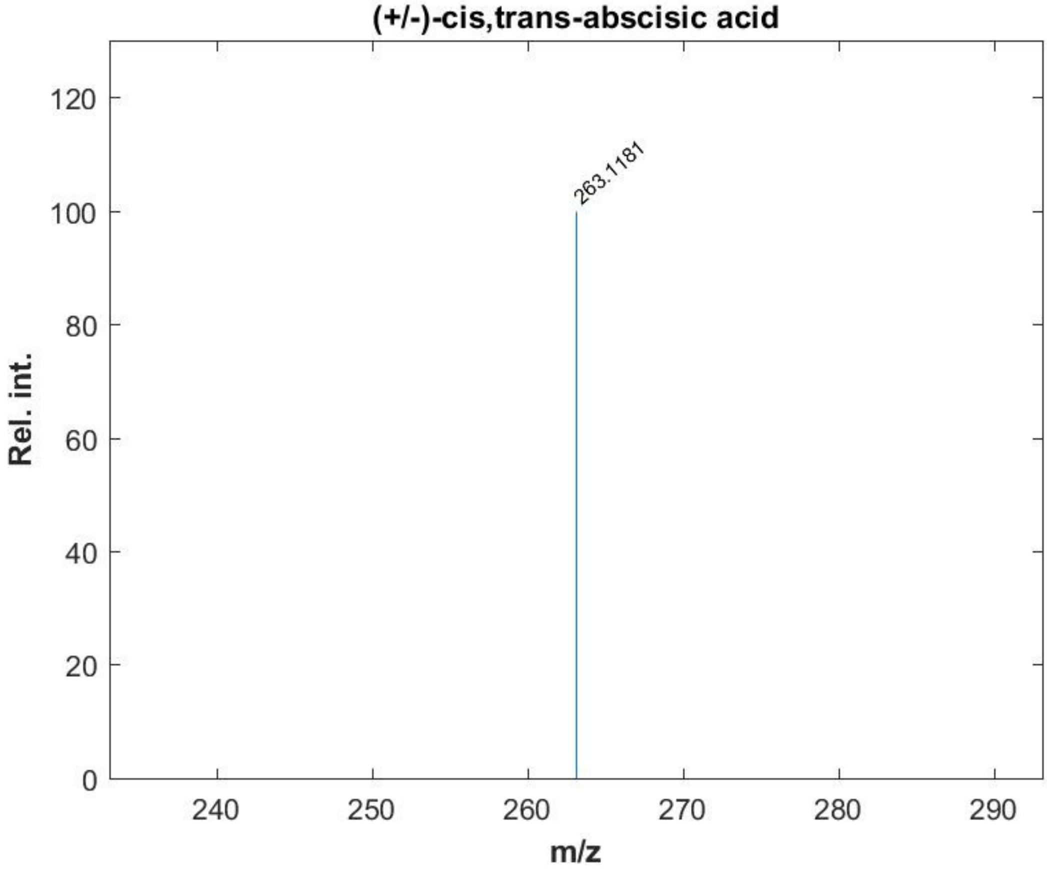

(+/-)-cis,trans-abscisic acid
Rel. int.
m/z
263.1181
0
20
40
60
80
100
120
240
250
260
270
280
290

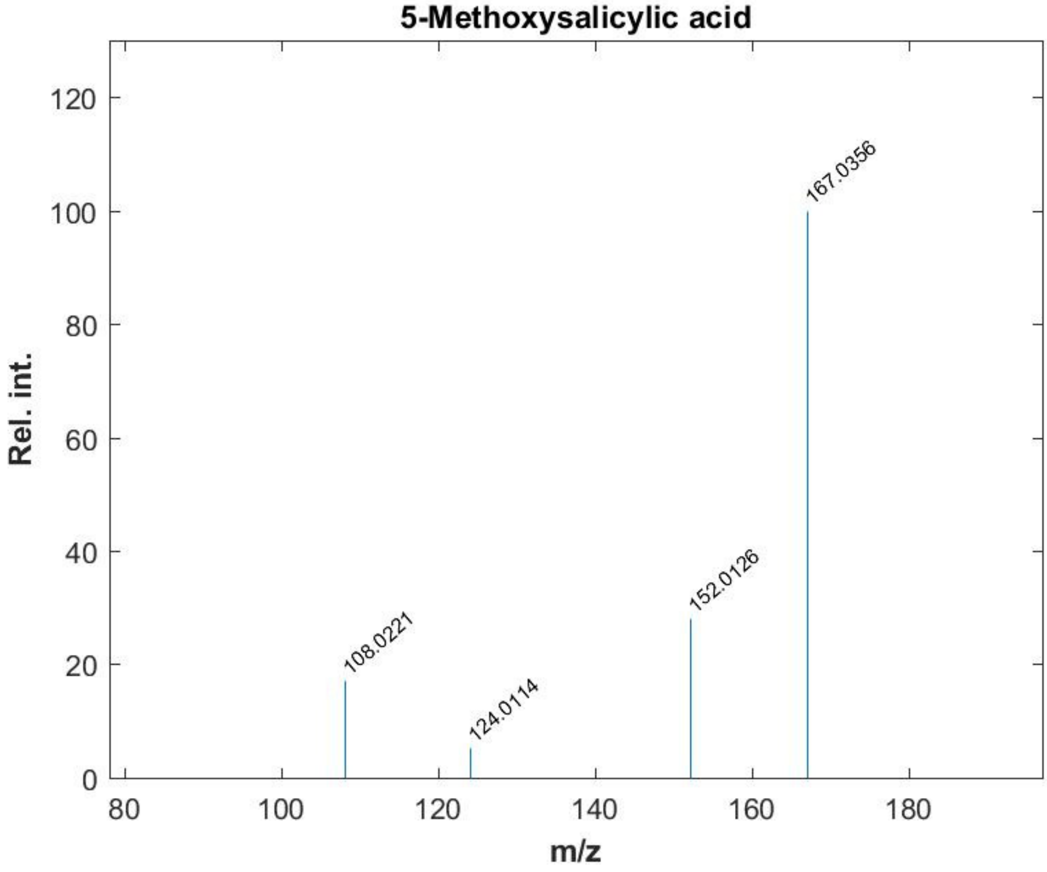

5-Methoxysalicylic acid
Rel. int.
m/z
108.0221
124.0114
152.0126
167.0356
0
20
40
60
80
100
120
80
100
120
140
160
180

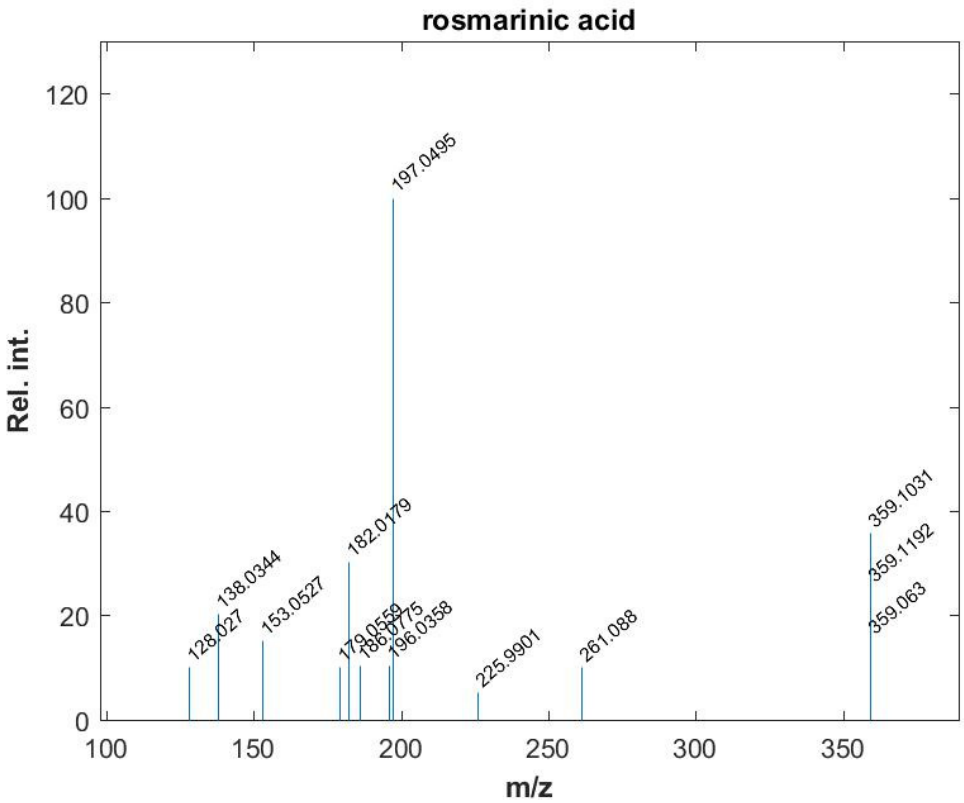
rosmarinic acid
Rel. int.
m/z
0
20
40
60
80
100
120
100
150
200
250
300
350
128.027
138.0344
153.0527
179.0559
182.0179
186.0775
196.0358
197.0495
225.9901
261.088
359.1031
359.1192
359.063

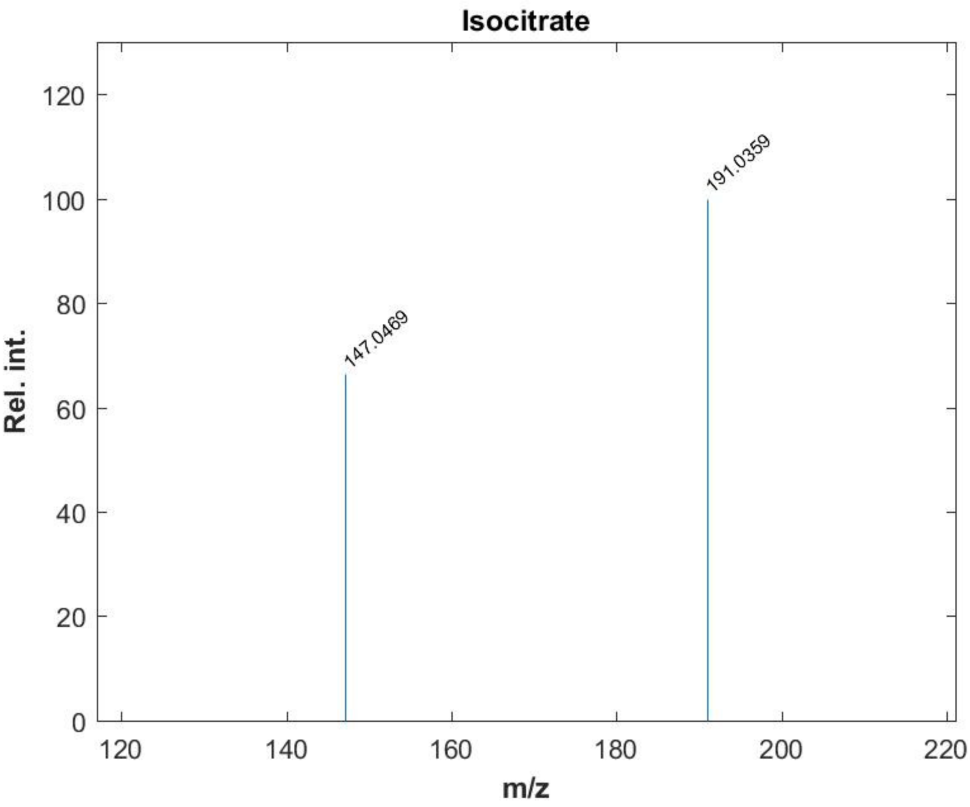
Isocitrate
Rel. int.
m/z
0
20
40
60
80
100
120
120
140
160
180
200
220
147.0469
191.0359

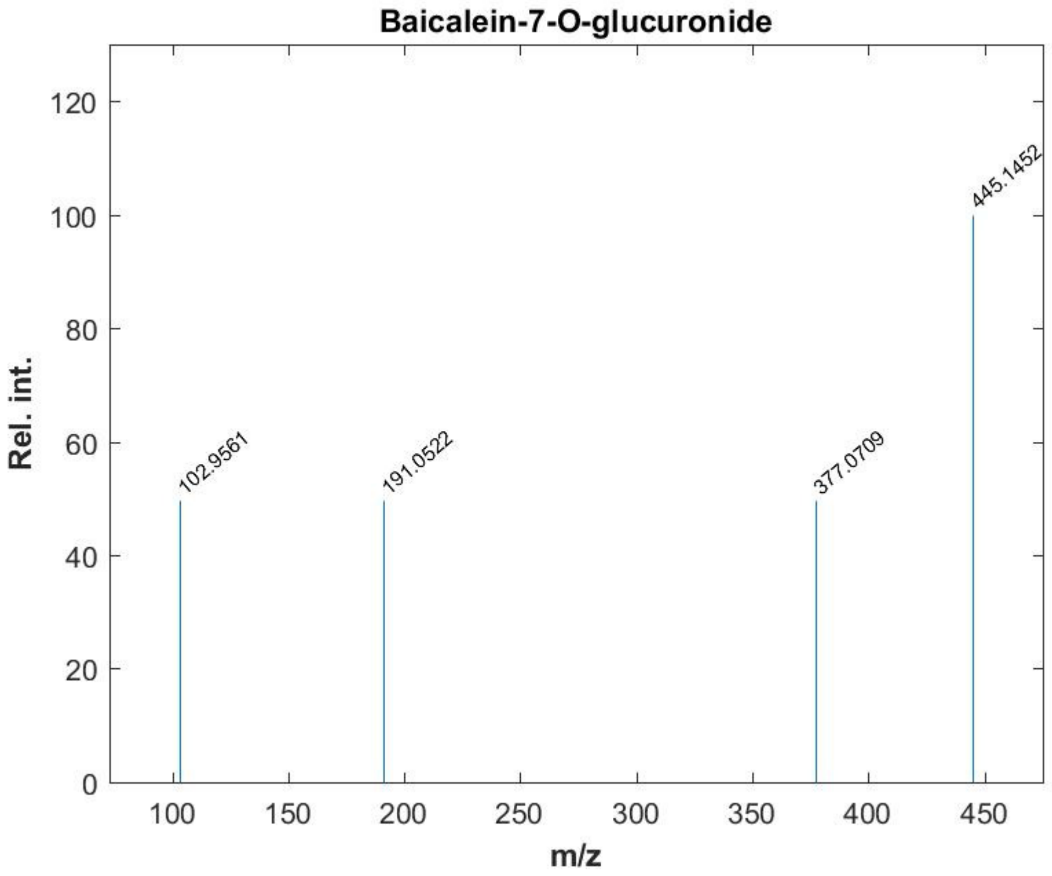
Baicalein-7-O-glucuronide
Rel. int.
m/z
0
20
40
60
80
100
120
100
150
200
250
300
350
400
450
102.9561
191.0522
377.0709
445.1452

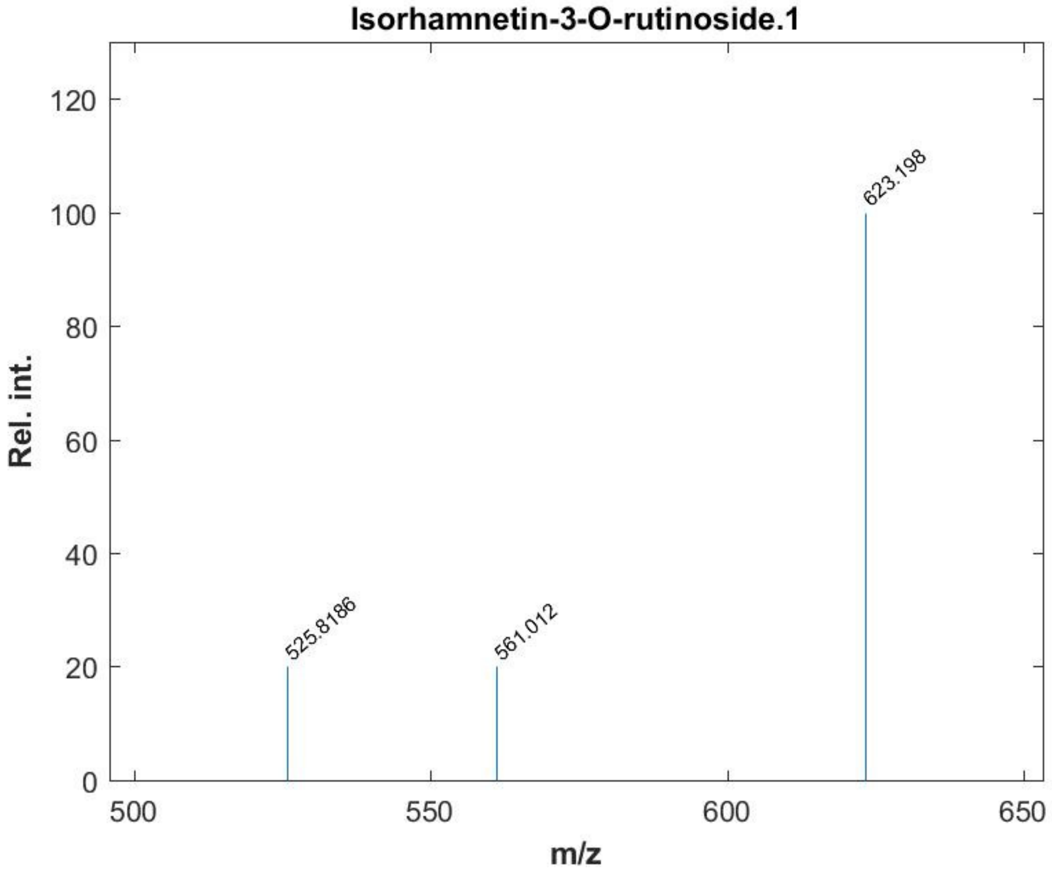
Isorhamnetin-3-O-rutinoside.1
Rel. int.
m/z
0
20
40
60
80
100
120
500
550
600
650
525.8186
561.012
623.198

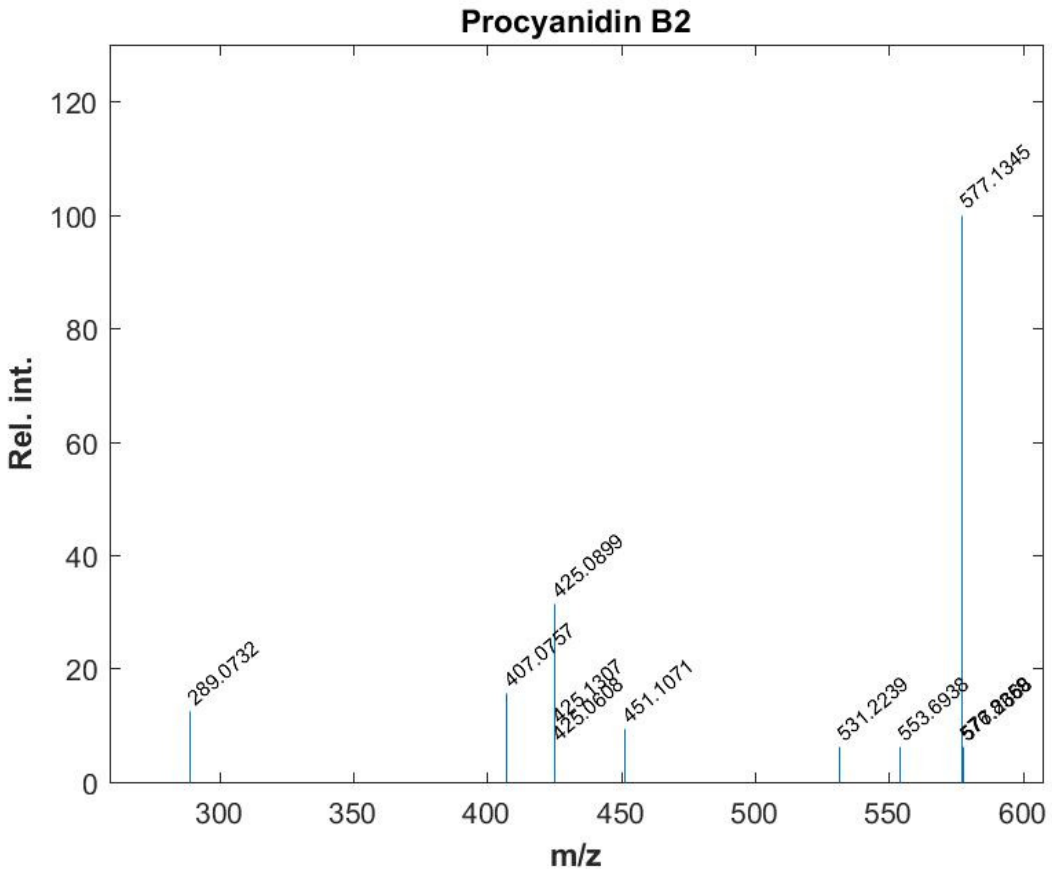
Procyanidin B2
Rel. int.
m/z
577.1345
425.0899
407.0757
425.1307
425.0608
451.1071
289.0732
531.2239
553.6938

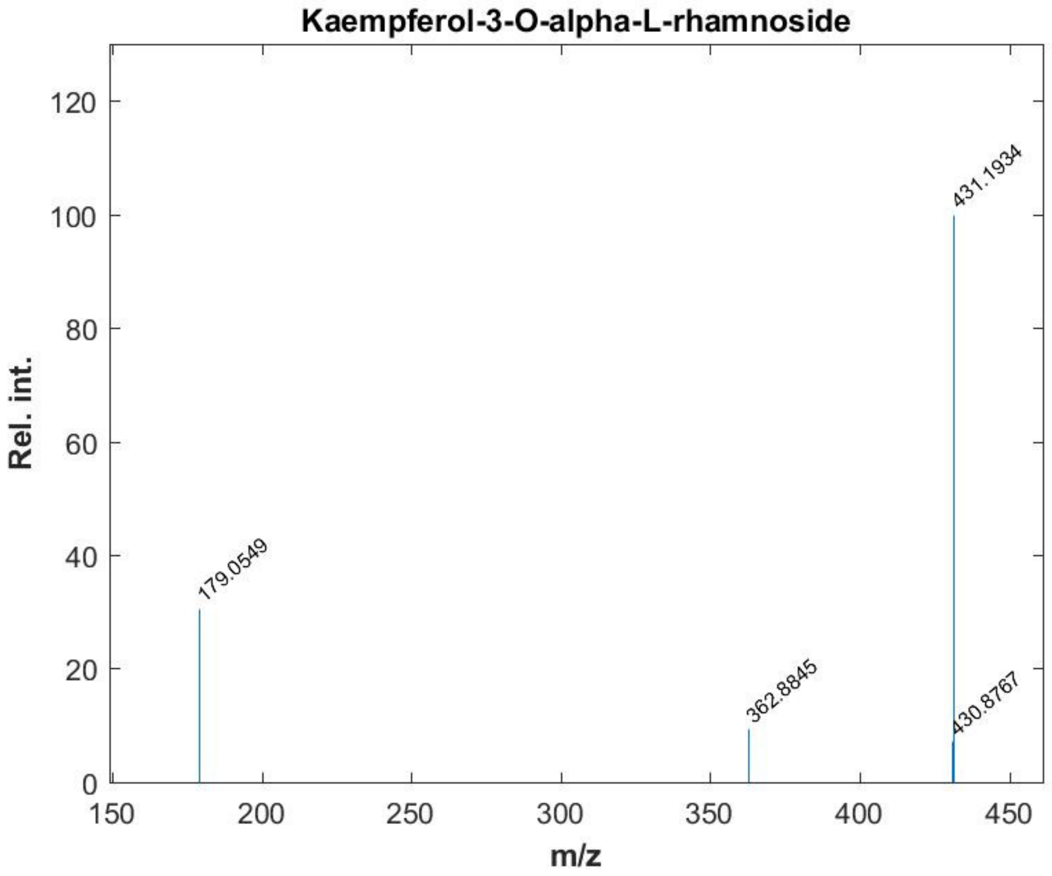
Kaempferol-3-O-alpha-L-rhamnoside
Rel. int.
m/z
431.1934
179.0549
362.8845
430.8767

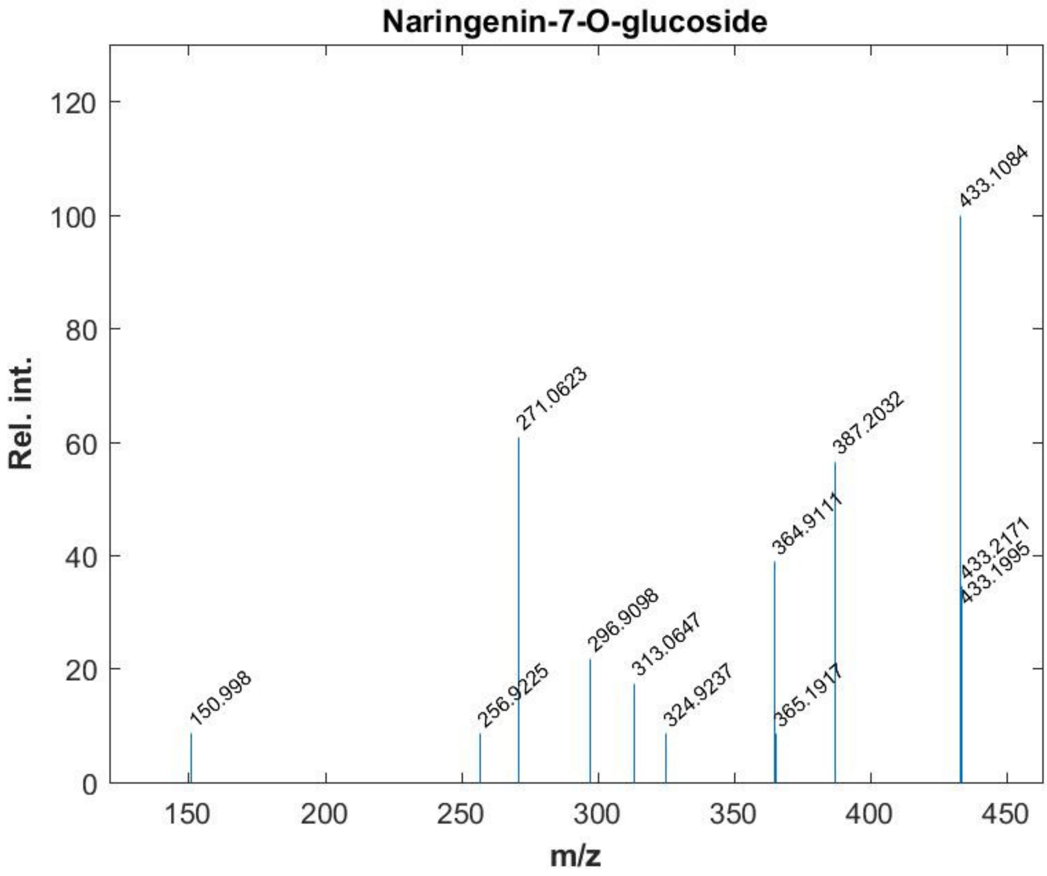

Naringenin-7-O-glucoside
Rel. int.
m/z
0
20
40
60
80
100
120
150
200
250
300
350
400
450
150.998
256.9225
271.0623
296.9098
313.0647
324.9237
364.9111
365.1917
387.2032
433.1084
433.2171
433.1995

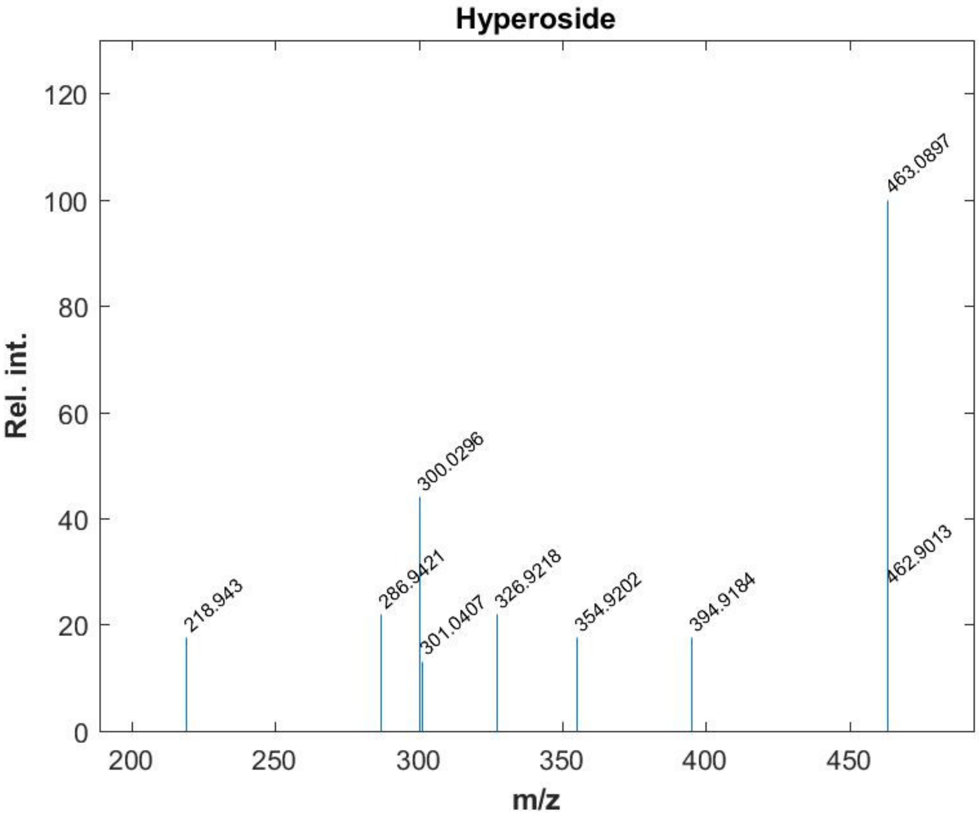
Hyperoside
Rel. int.
m/z
0
20
40
60
80
100
120
200
250
300
350
400
450
218.943
286.9421
300.0296
301.0407
326.9218
354.9202
394.9184
462.9013
463.0897

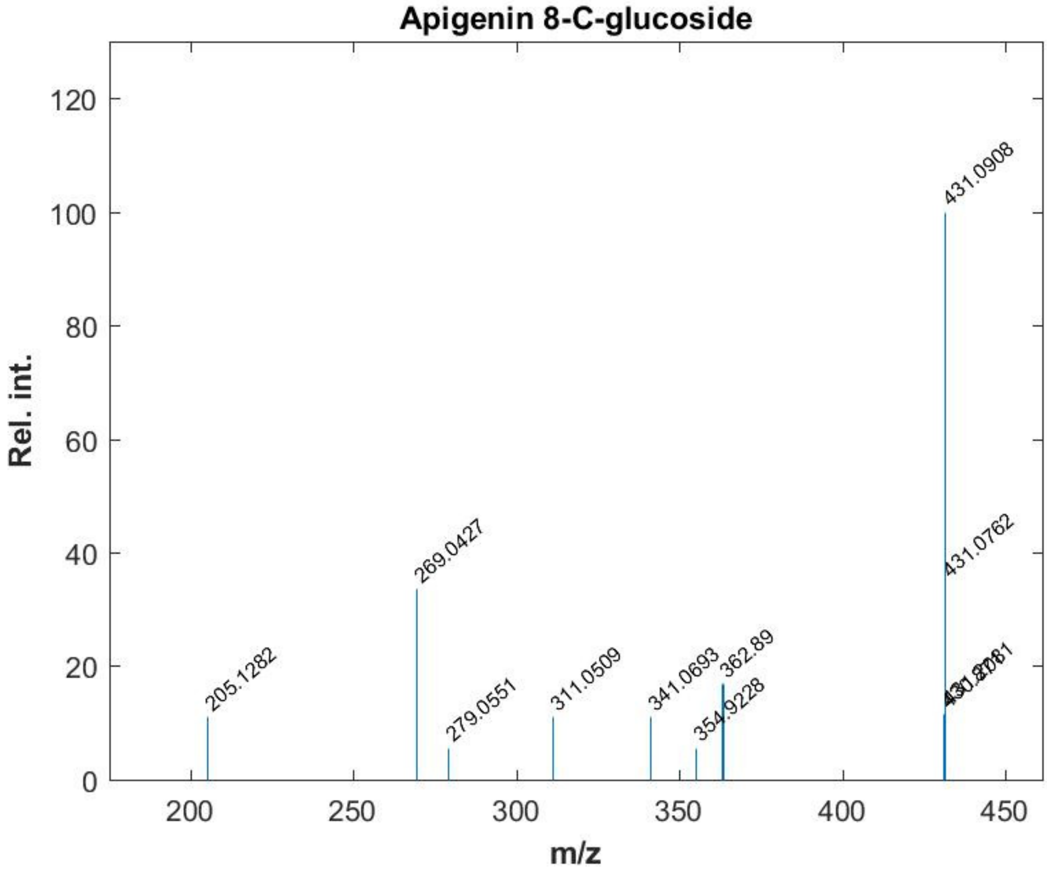
Apigenin 8-C-glucoside
Rel. int.
m/z
0
20
40
60
80
100
120
200
250
300
350
400
450
205.1282
269.0427
279.0551
311.0509
341.0693
354.9228
362.89
431.0762
431.0908

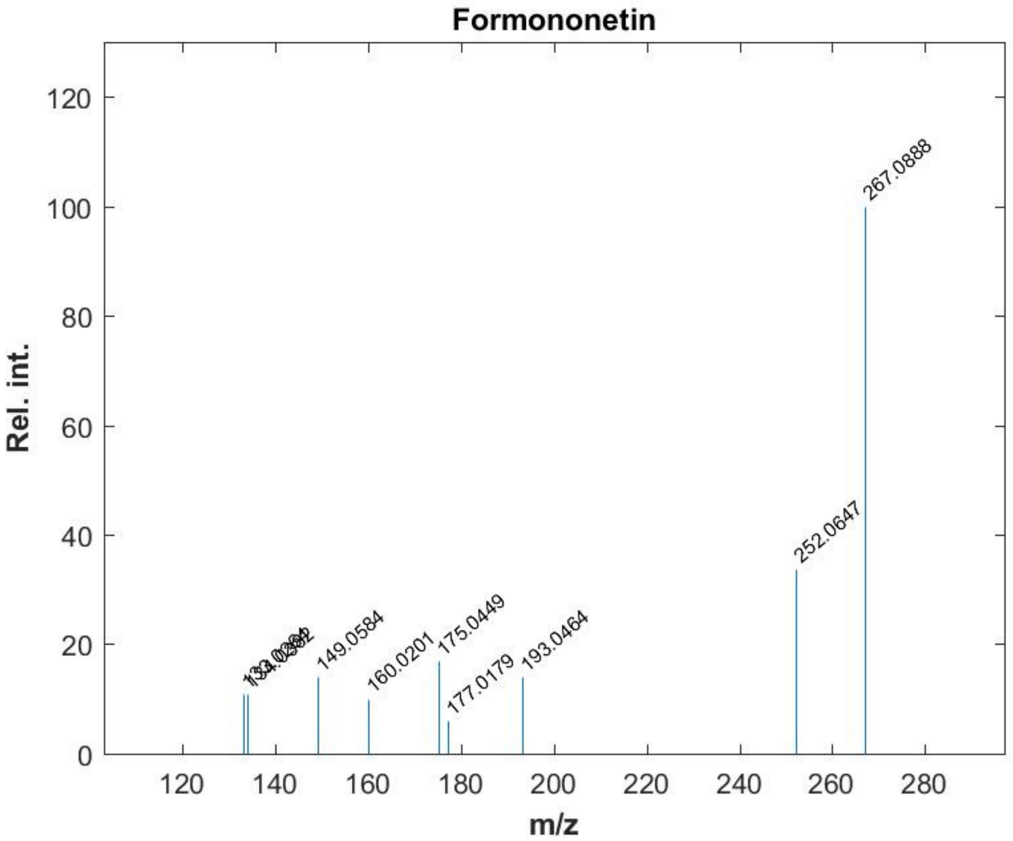
Formononetin
Rel. int.
m/z
267.0888
252.0647
149.0584
160.0201
175.0449
177.0179
193.0464
0
20
40
60
80
100
120
120
140
160
180
200
220
240
260
280

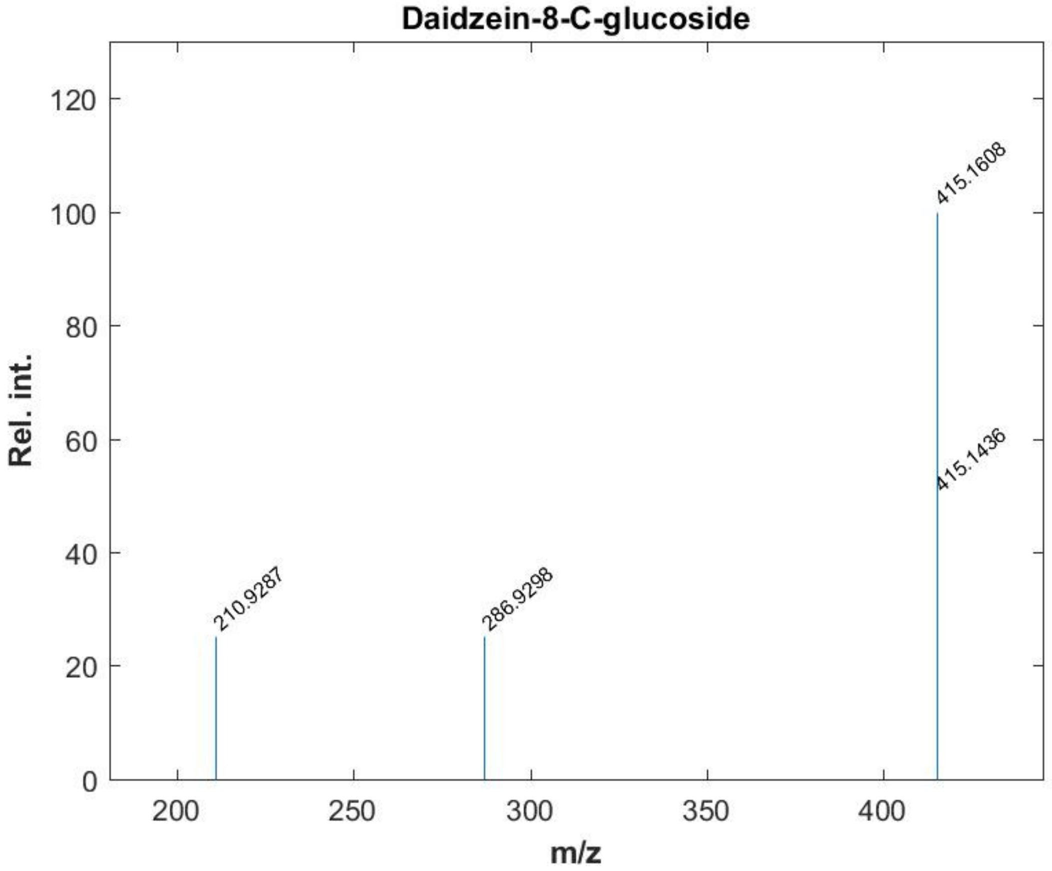
Daidzein-8-C-glucoside
Rel. int.
m/z
415.1608
415.1436
210.9287
286.9298
0
20
40
60
80
100
120
200
250
300
350
400

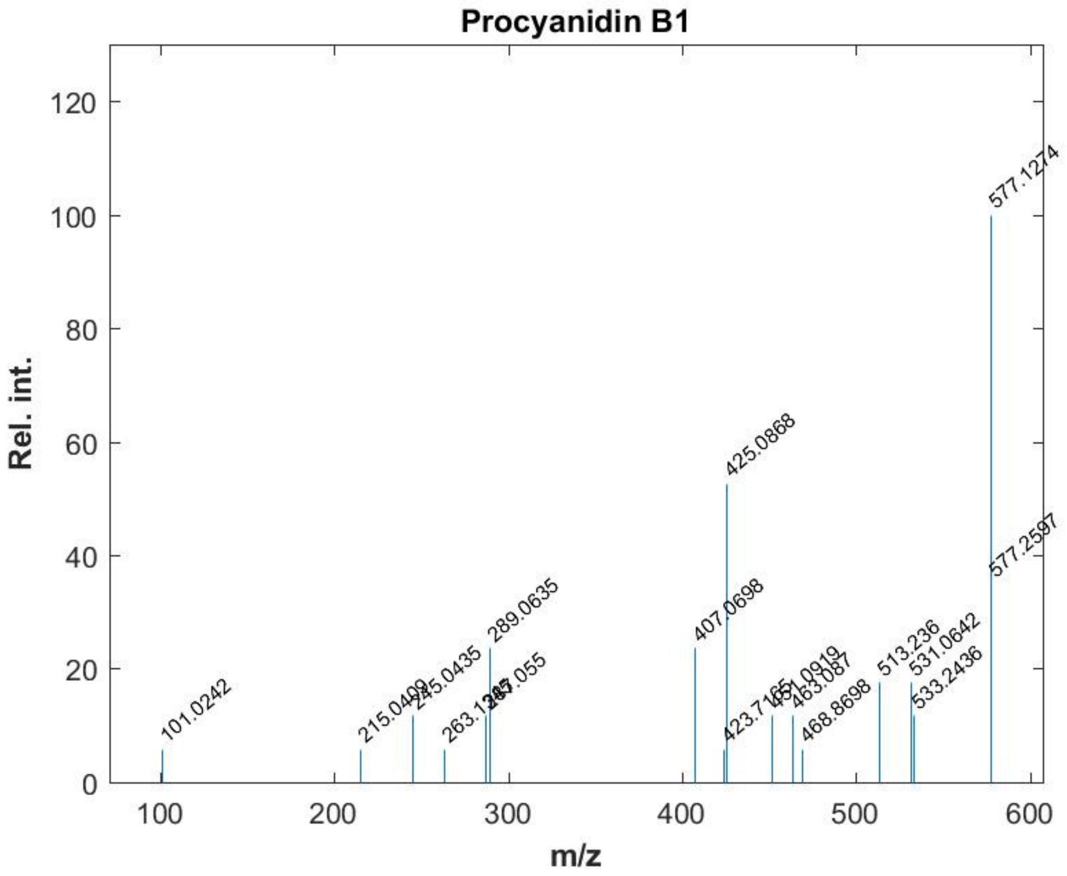
Procyanidin B1
Rel. int.
m/z
0
20
40
60
80
100
120
100
200
300
400
500
600
101.0242
245.0435
289.0635
407.0698
425.0868
463.087
468.8698
513.236
531.0642
533.2436
577.1274
577.2597

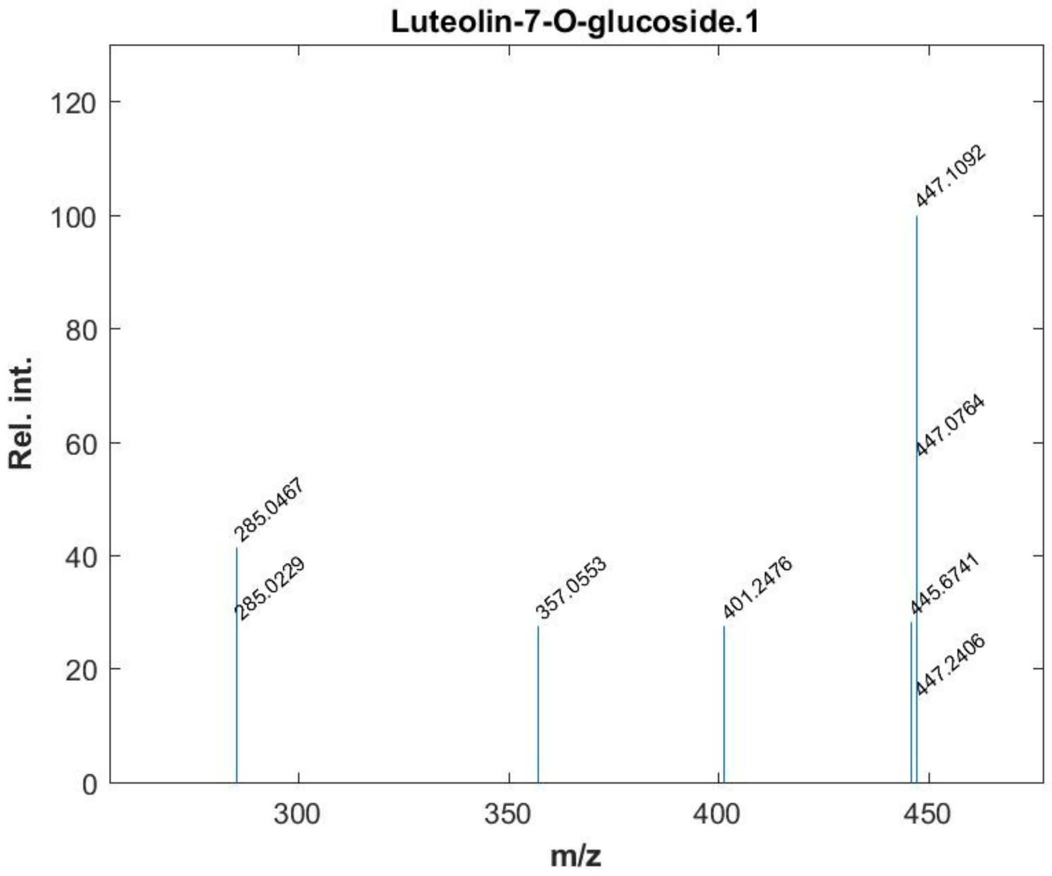
Luteolin-7-O-glucoside.1
Rel. int.
m/z
447.1092
447.0764
285.0467
285.0229
357.0553
401.2476
445.6741
447.2406

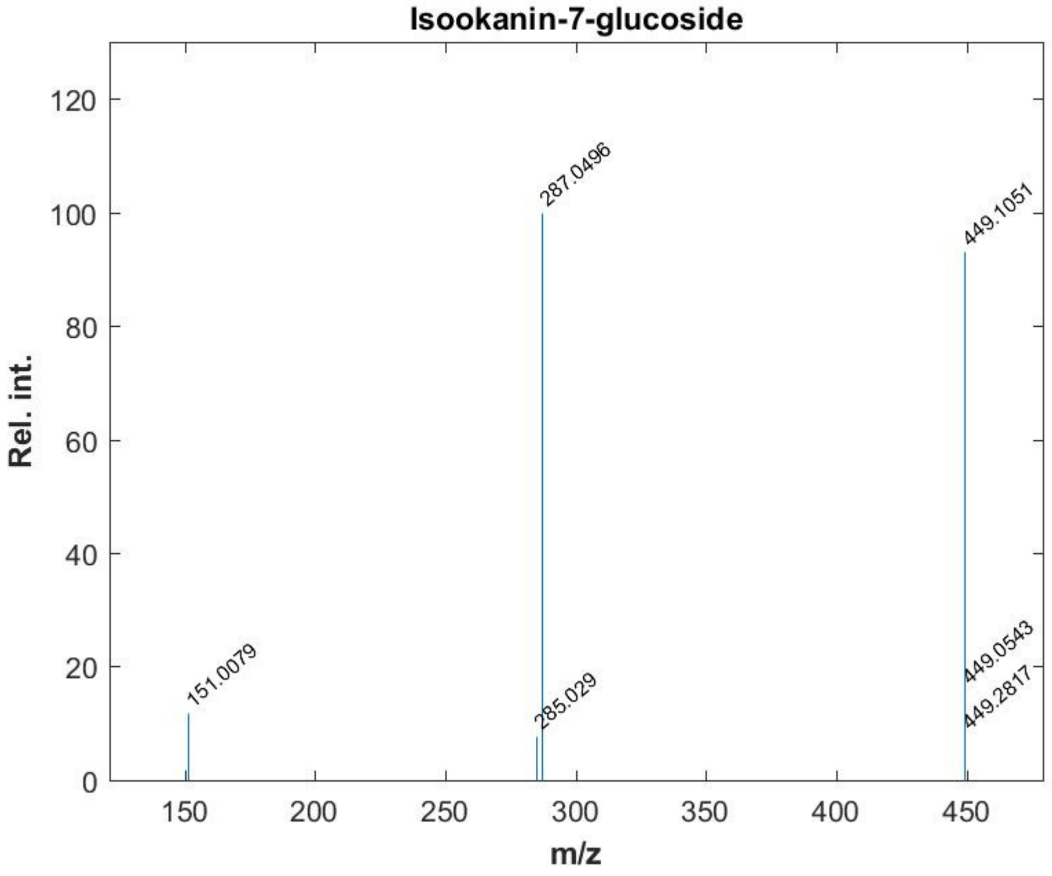
Isookanin-7-glucoside
Rel. int.
m/z
287.0496
449.1051
151.0079
285.029
449.0543
449.2817

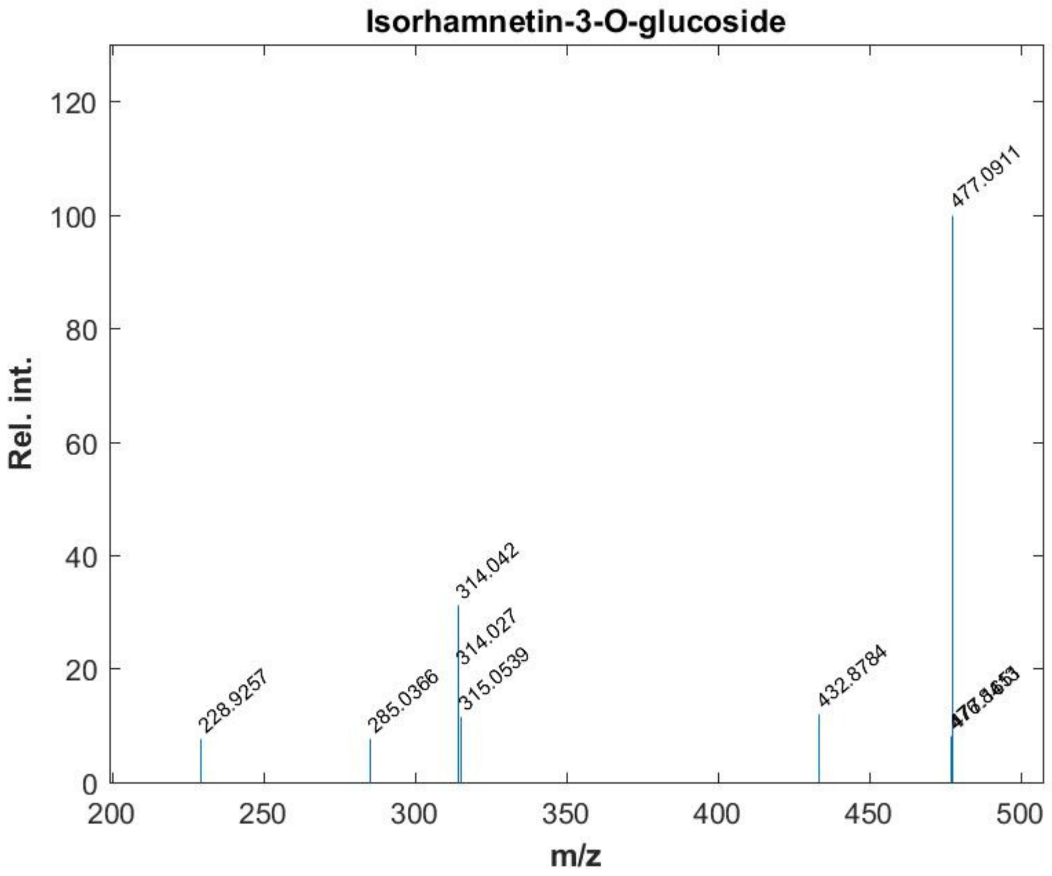
Isorhamnetin-3-O-glucoside
Rel. int.
m/z
0
20
40
60
80
100
120
200
250
300
350
400
450
500
228.9257
285.0366
314.042
314.027
315.0539
432.8784
477.0911

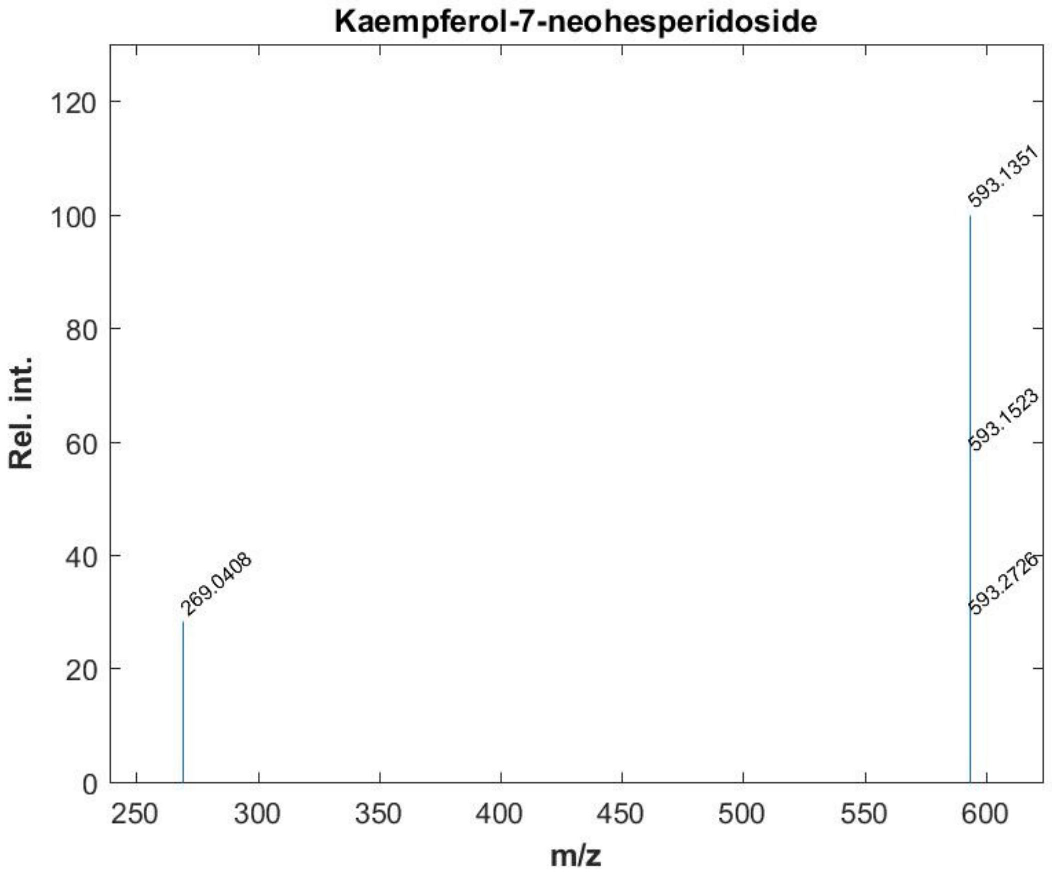
Kaempferol-7-neohesperidoside
Rel. int.
m/z
0
20
40
60
80
100
120
250
300
350
400
450
500
550
600
269.0408
593.1351
593.1523
593.2726

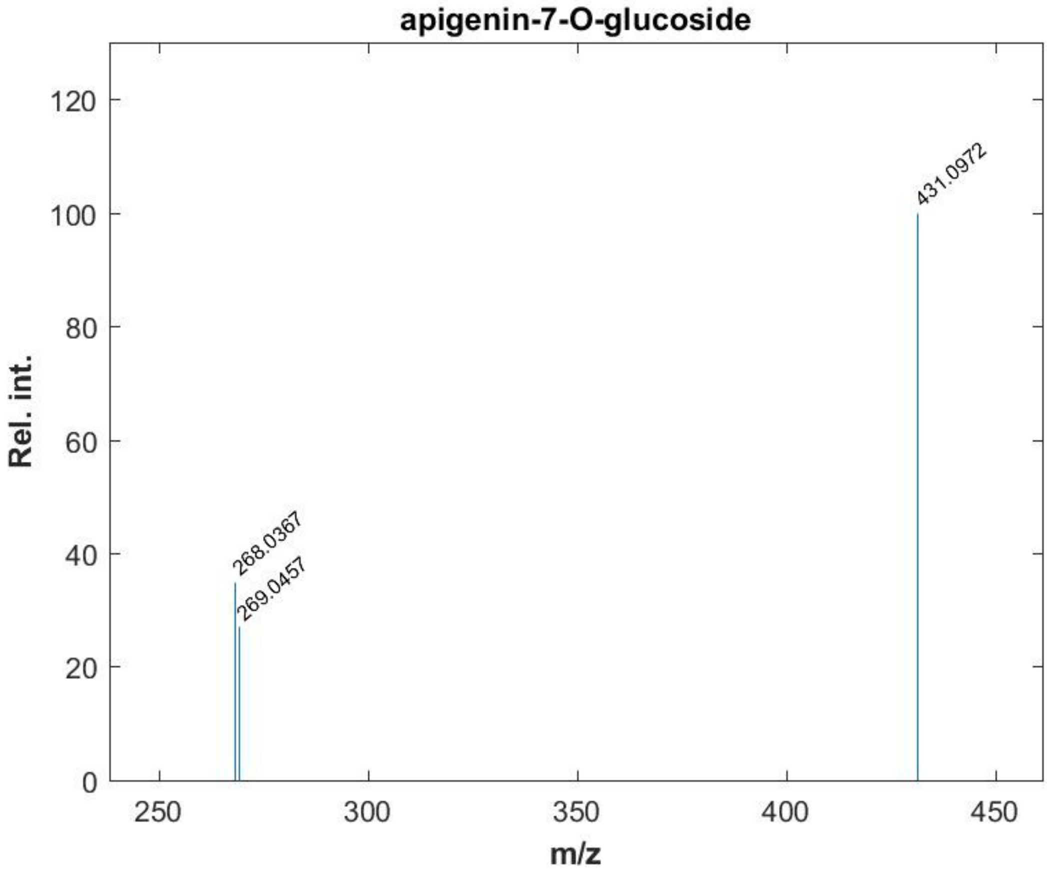
apigenin-7-O-glucoside
Rel. int.
m/z
0
20
40
60
80
100
120
250
300
350
400
450
268.0367
269.0457
431.0972

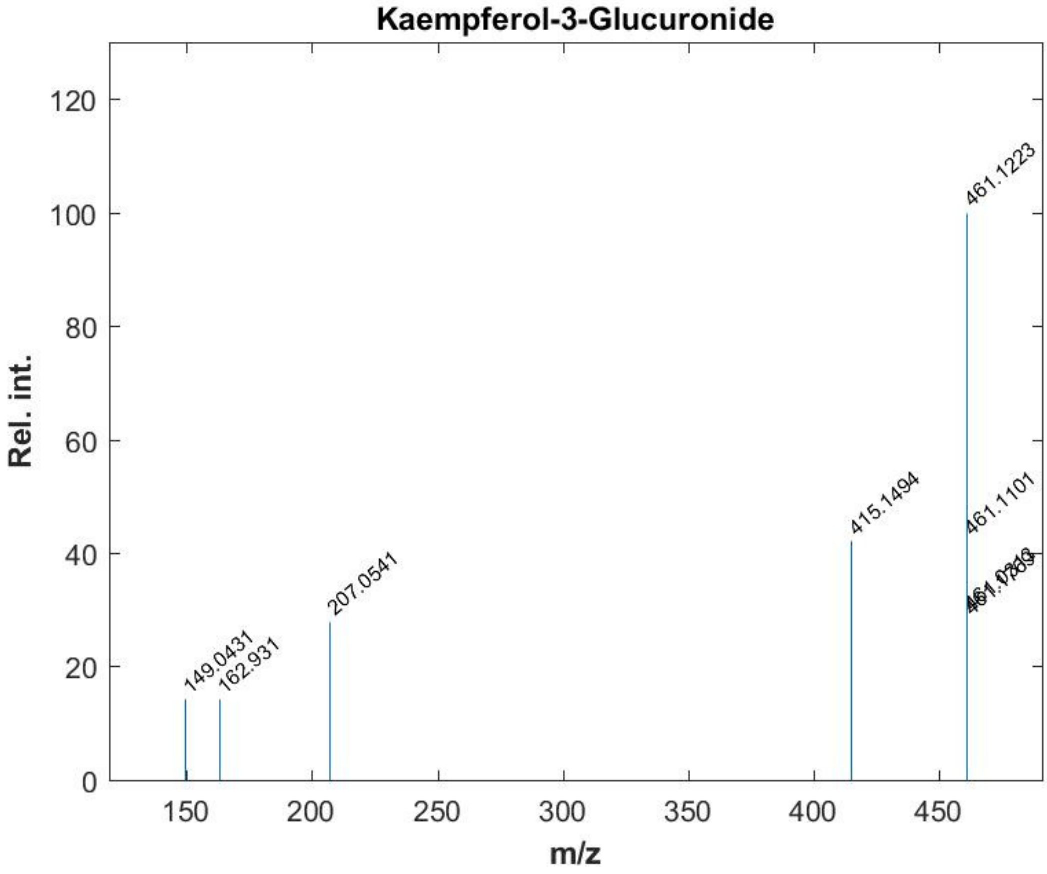
Kaempferol-3-Glucuronide
Rel. int.
m/z
0
20
40
60
80
100
120
150
200
250
300
350
400
450
149.0431
162.931
207.0541
415.1494
461.1223
461.1101

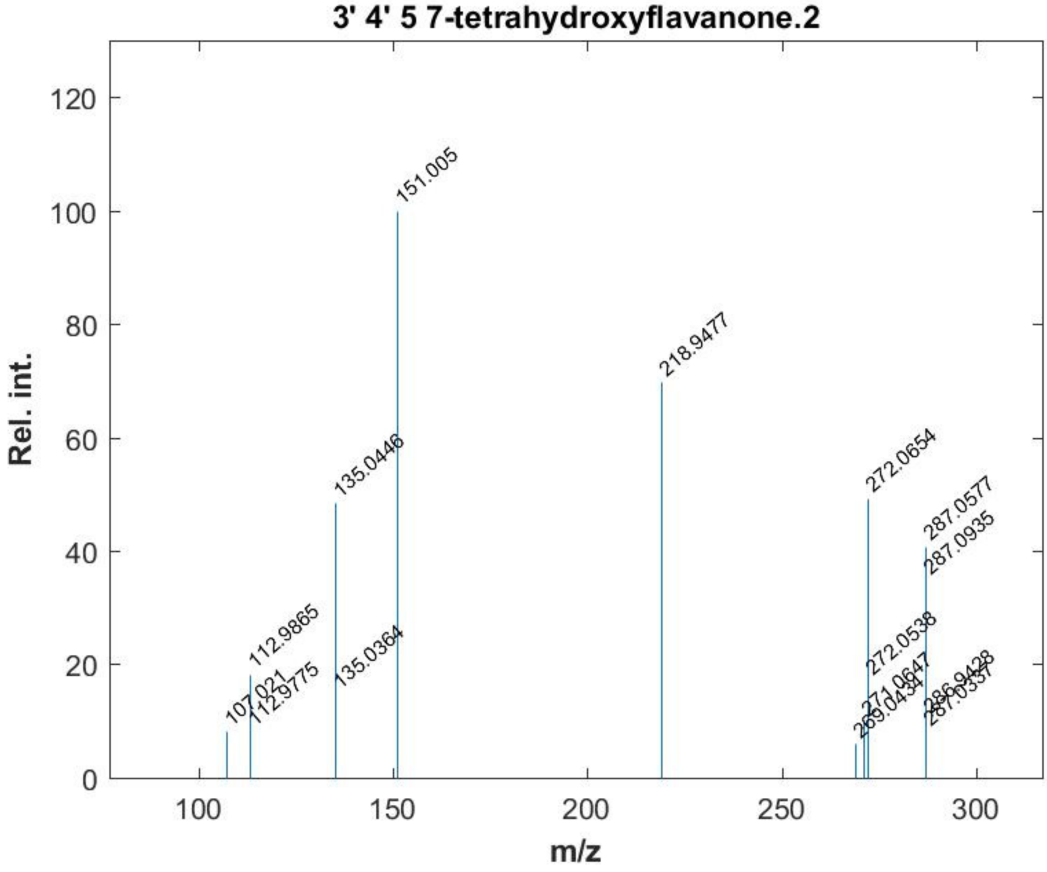
3' 4' 5 7-tetrahydroxyflavanone.2
Rel. int.
m/z
0
20
40
60
80
100
120
100
150
200
250
300
151.005
218.9477
135.0446
272.0654
287.0577
287.0935
112.9865
135.0364
272.0538
107.021
112.9775
271.0647
269.0434
286.9428
287.0337

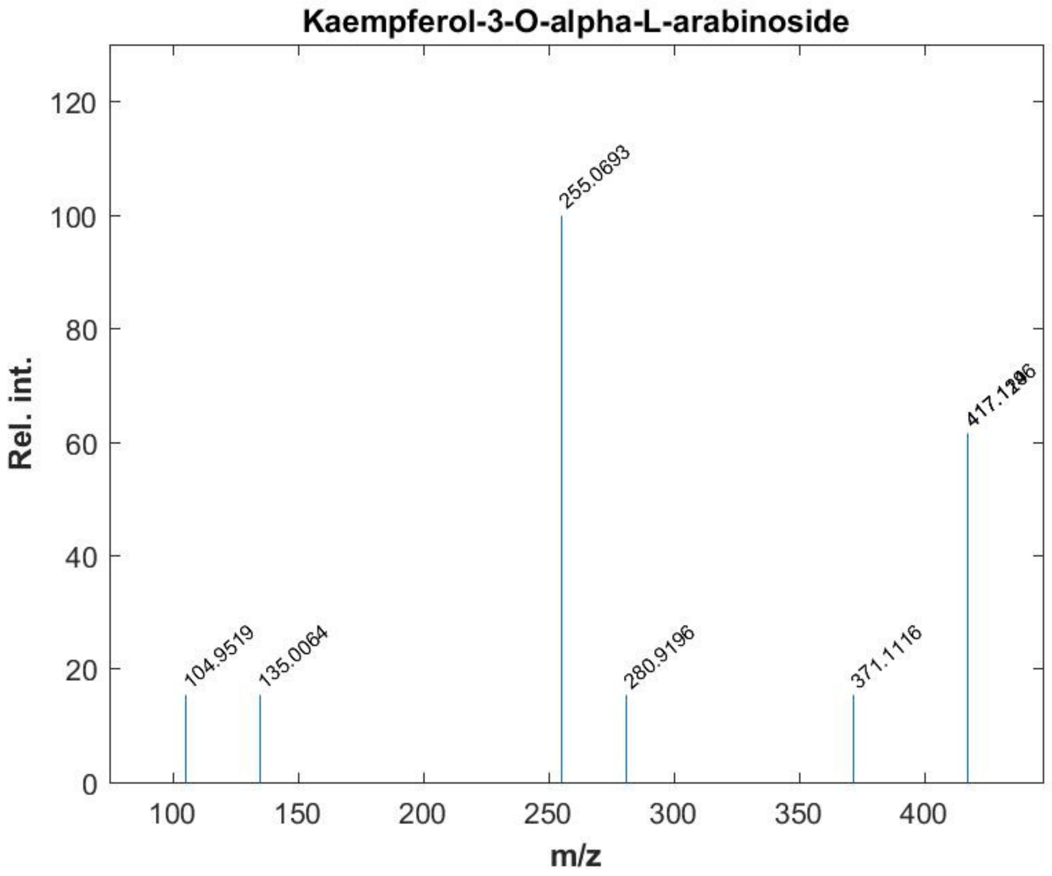

Kaempferol-3-O-alpha-L-arabinoside
Rel. int.
m/z
104.9519
135.0064
255.0693
280.9196
371.1116

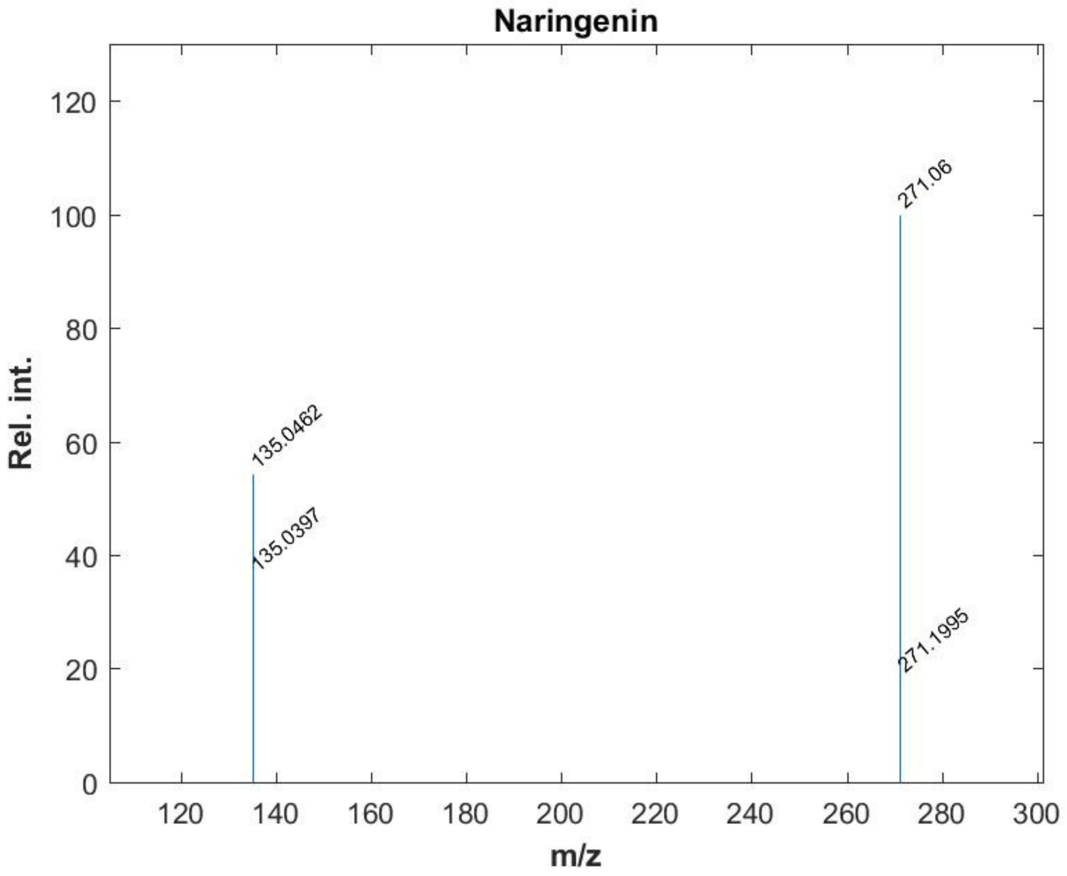

Naringenin
Rel. int.
m/z
135.0462
135.0397
271.06
271.1995

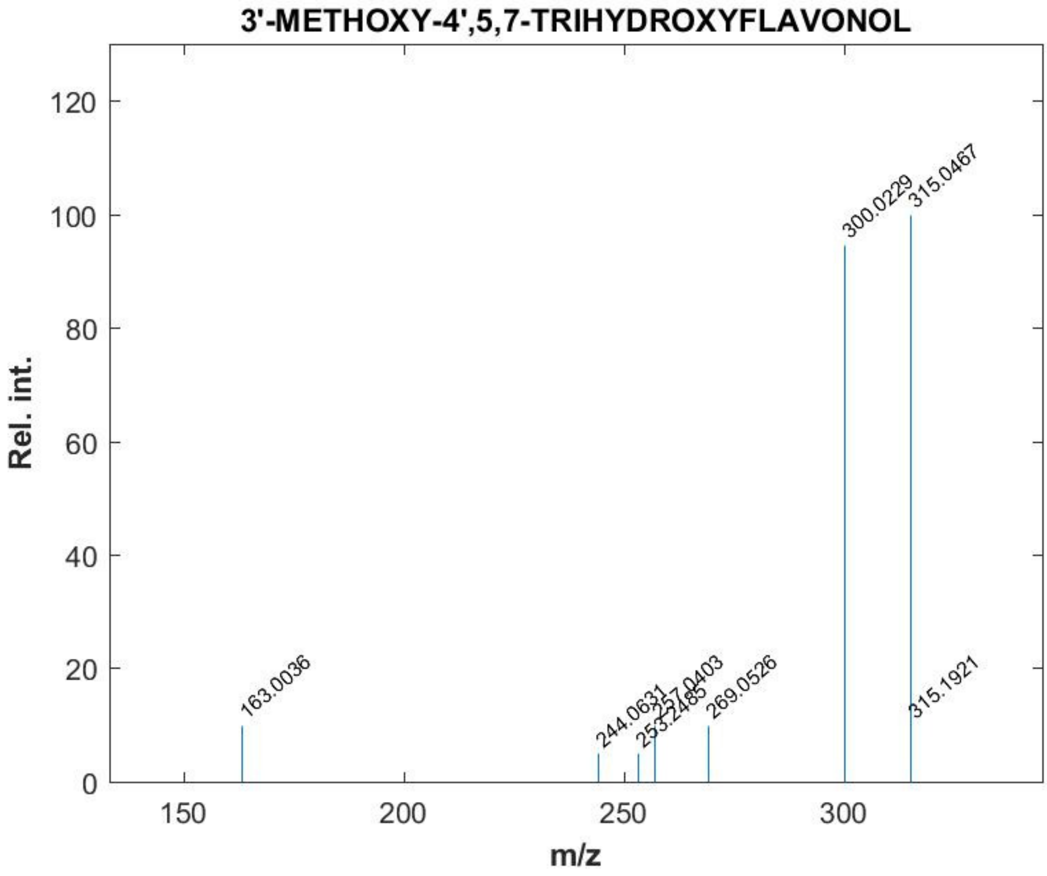

3'-METHOXY-4',5,7-TRIHYDROXYFLAVONOL
Rel. int.
m/z
0
20
40
60
80
100
120
150
200
250
300
163.0036
244.0631
253.2485
257.0403
269.0526
300.0229
315.0467
315.1921

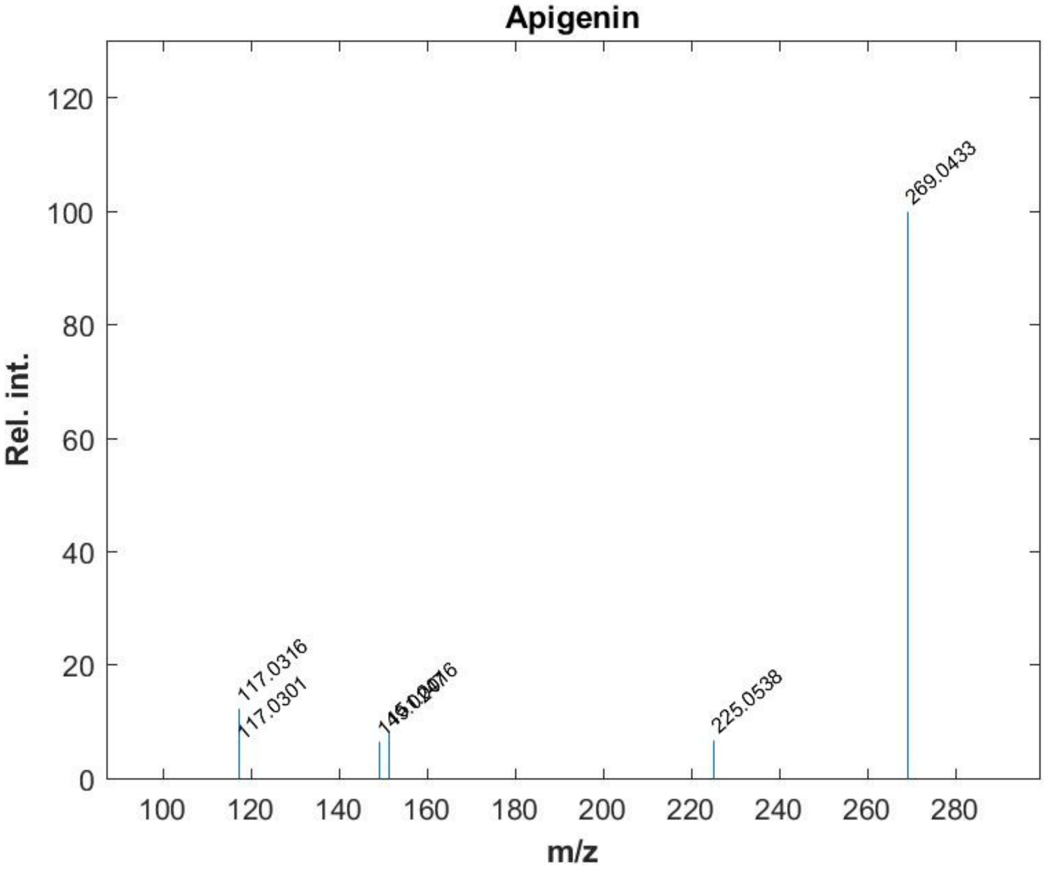
Apigenin
Rel. int.
m/z
117.0316
117.0301
225.0538
269.0433
0
20
40
60
80
100
120
100
120
140
160
180
200
220
240
260
280

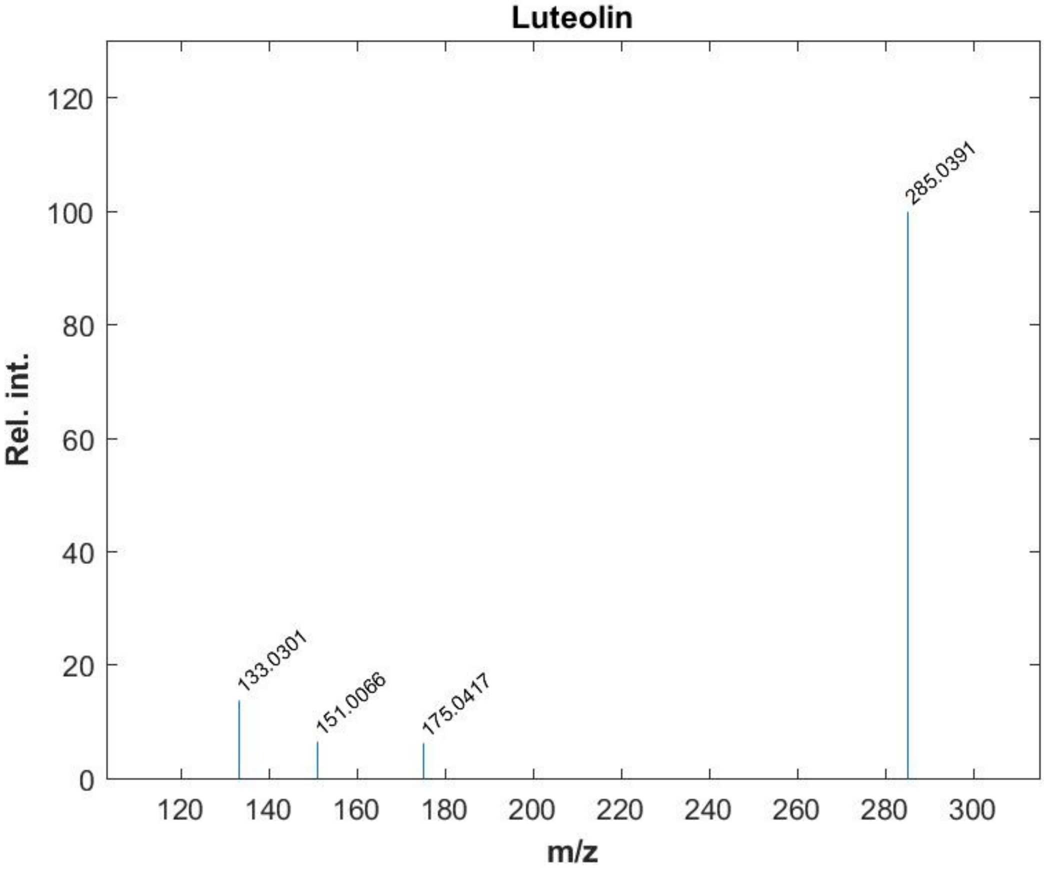
Luteolin
Rel. int.
m/z
133.0301
151.0066
175.0417
285.0391
0
20
40
60
80
100
120
120
140
160
180
200
220
240
260
280
300

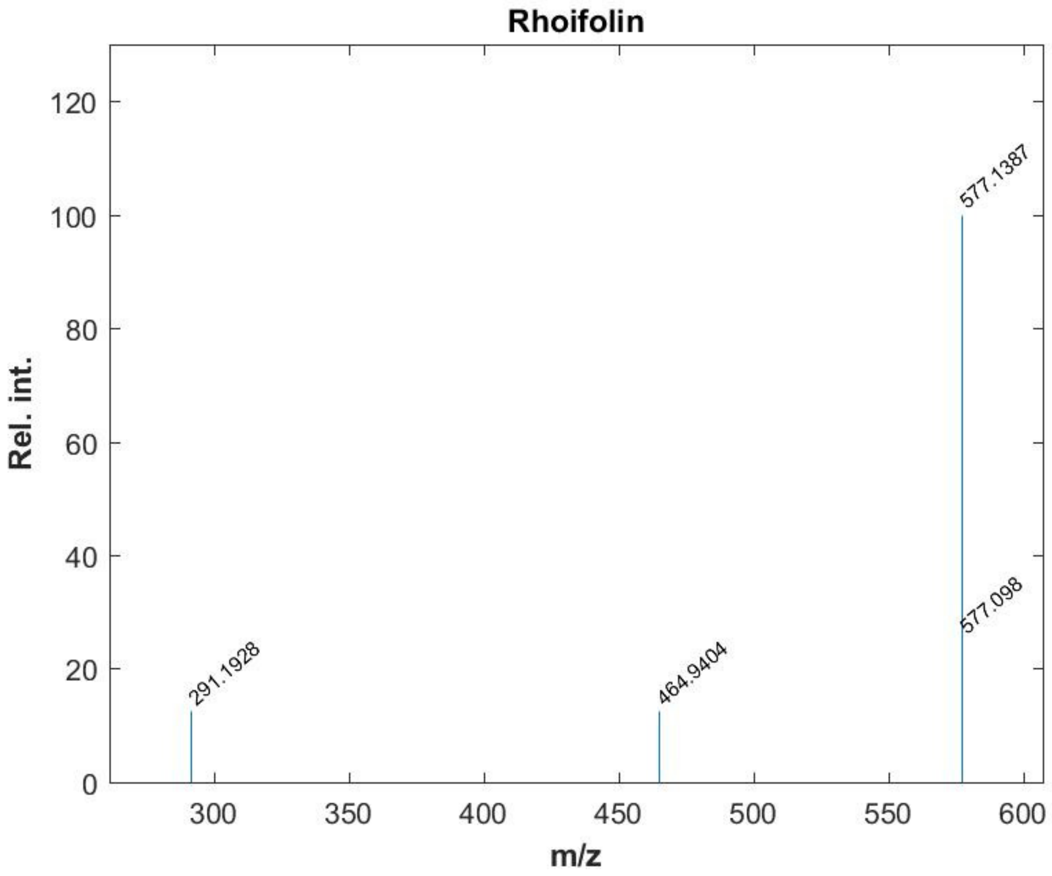
Rhoifolin
Rel. int.
m/z
0
20
40
60
80
100
120
300
350
400
450
500
550
600
291.1928
464.9404
577.1387
577.098

3 5 7-trihydroxy-4'-methoxyflavone

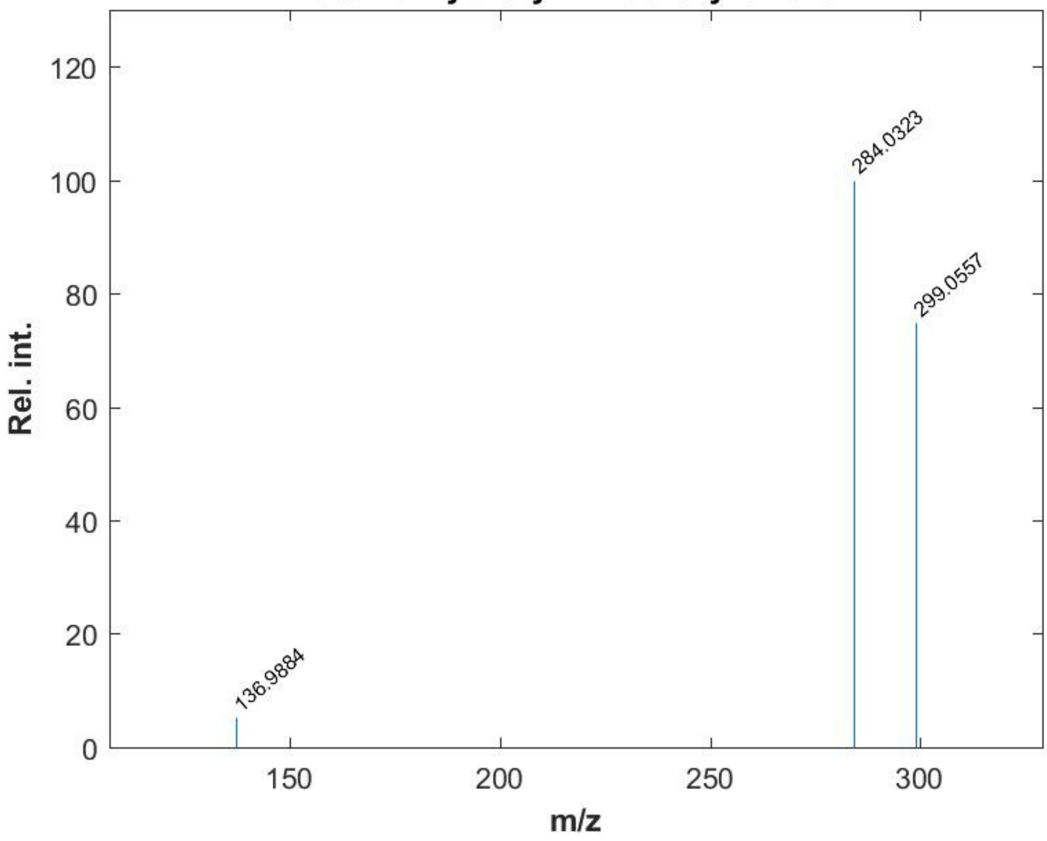

3'-METHOXY-4',5,7-TRIHYDROXYFLAVONOL

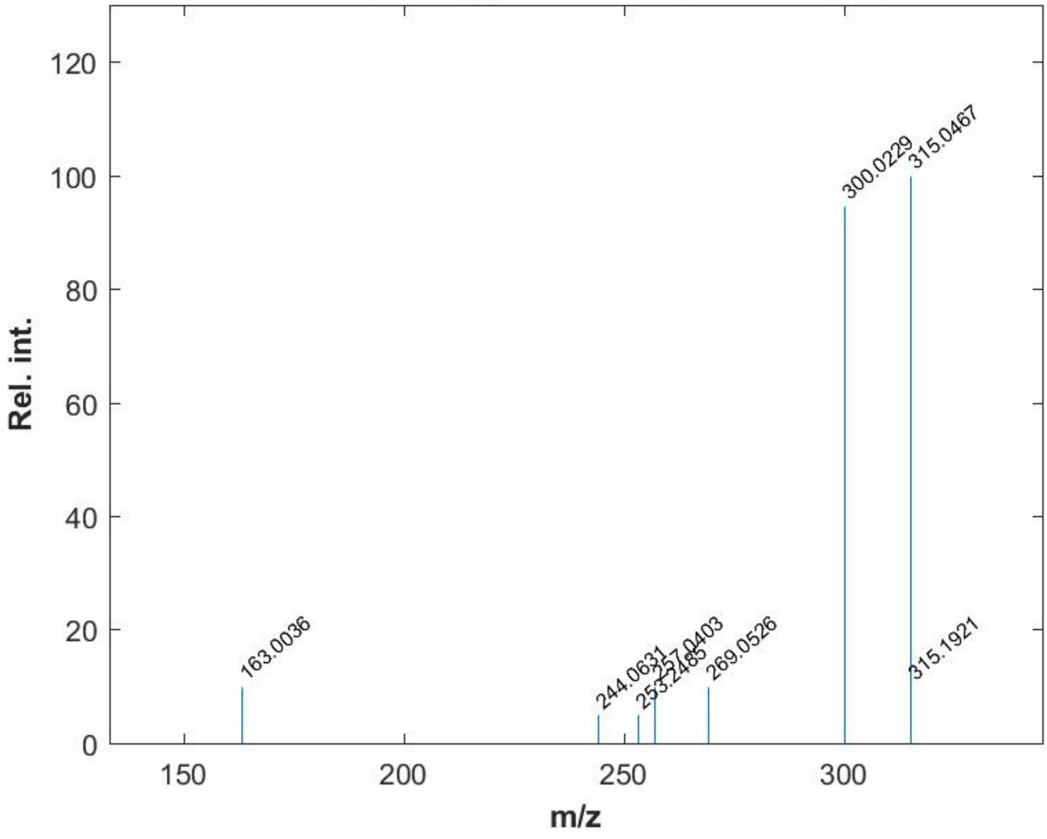

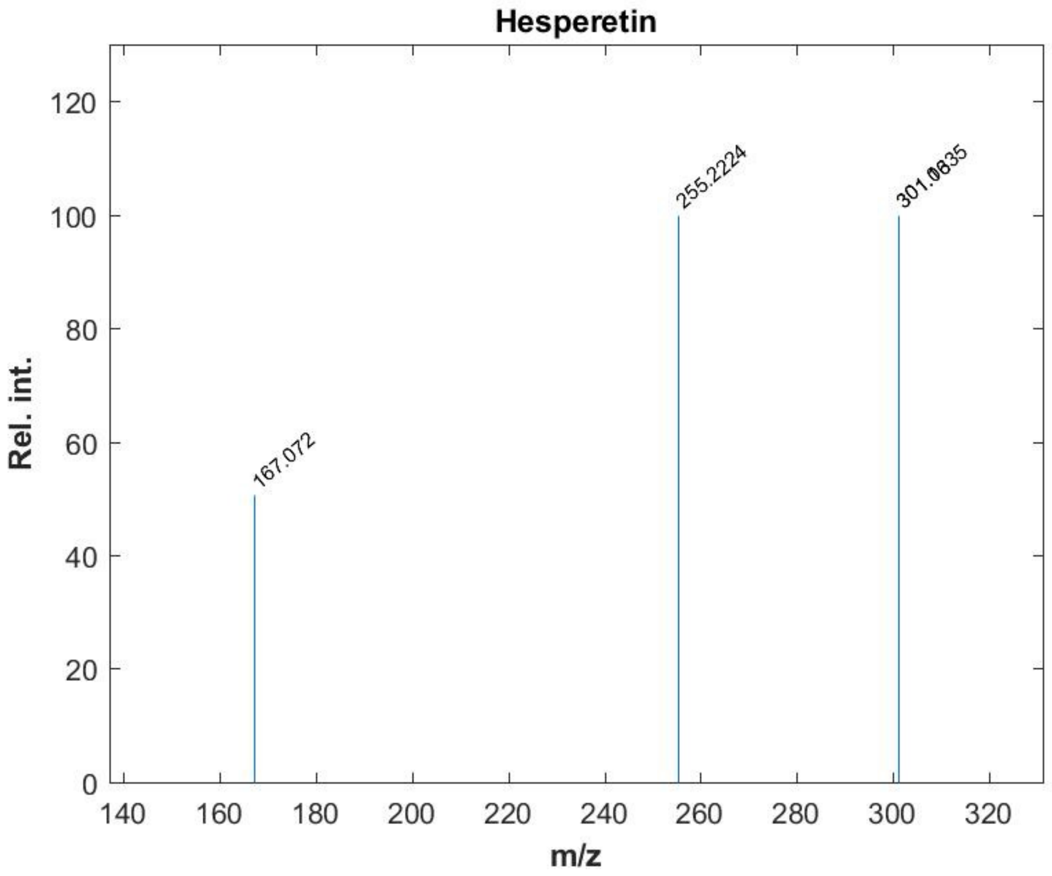
Hesperetin
Rel. int.
m/z
167.072
255.2224
301.0835
0
20
40
60
80
100
120
140
160
180
200
220
240
260
280
300
320

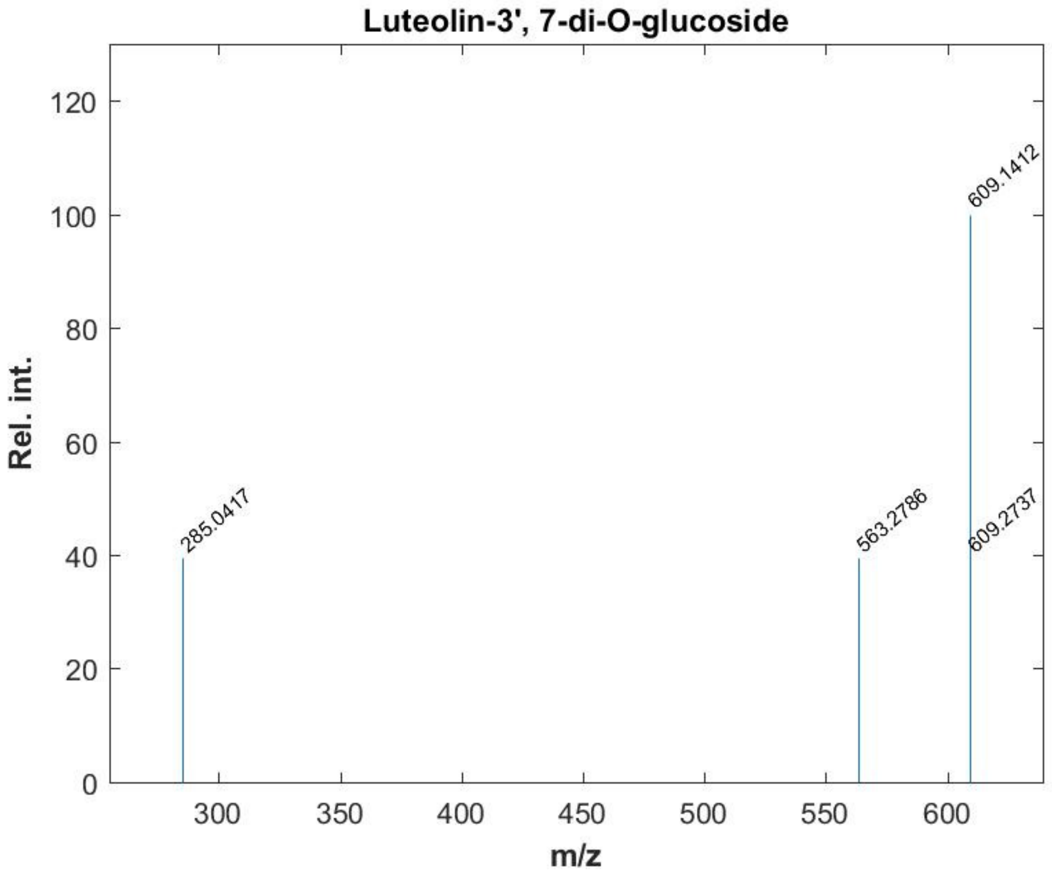
Luteolin-3', 7-di-O-glucoside
Rel. int.
m/z
285.0417
563.2786
609.1412
609.2737
0
20
40
60
80
100
120
300
350
400
450
500
550
600

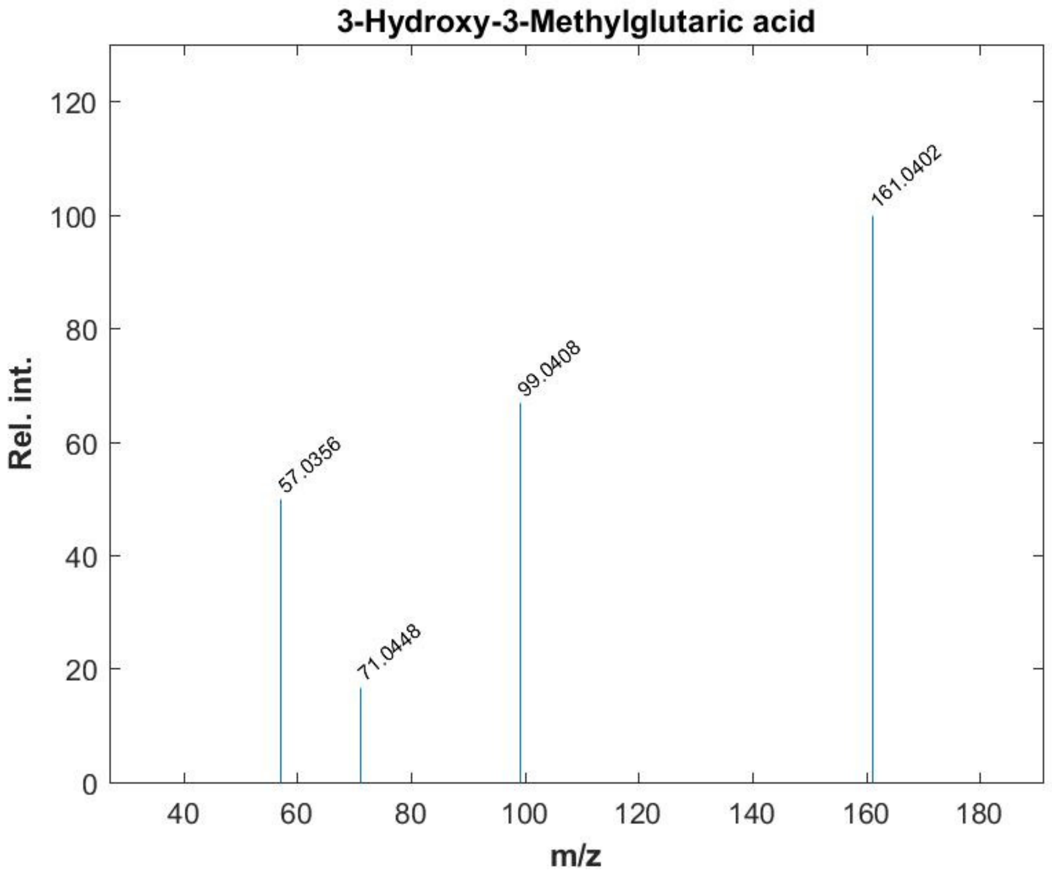

3-Hydroxy-3-Methylglutaric acid
Rel. int.
m/z
0
20
40
60
80
100
120
40
60
80
100
120
140
160
180
57.0356
71.0448
99.0408
161.0402

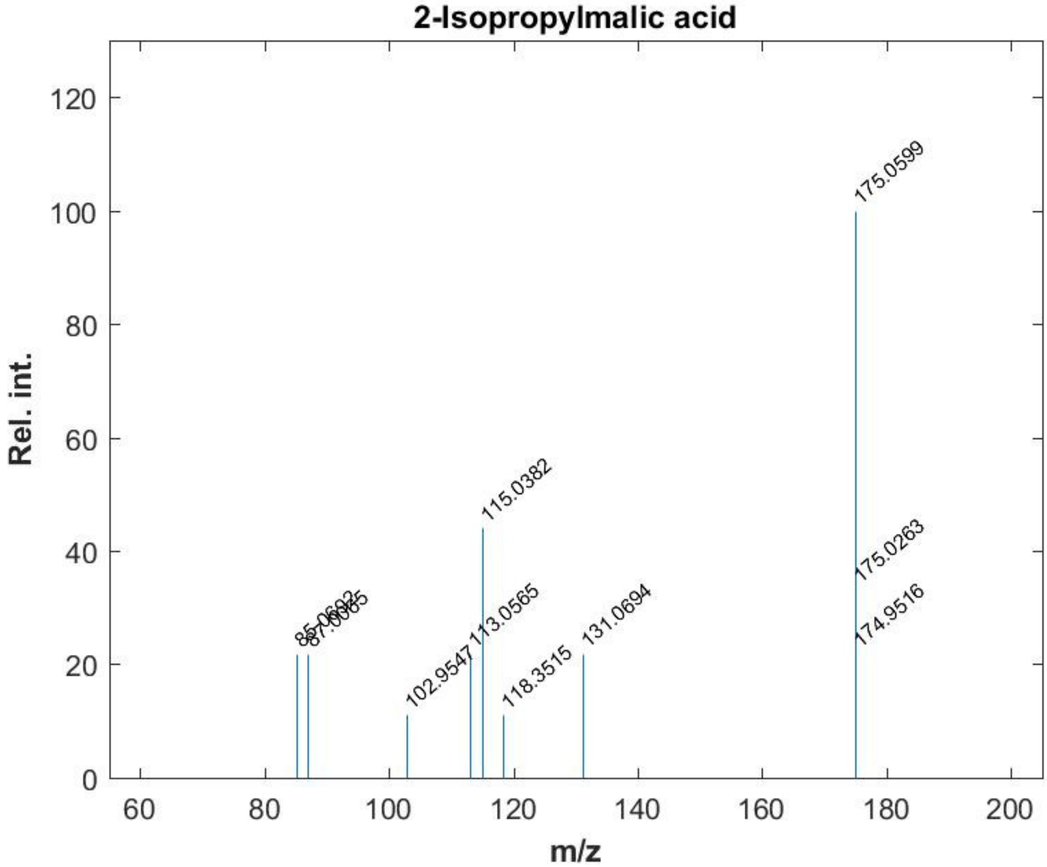
2-Isopropylmalic acid
Rel. int.
m/z
175.0599
115.0382
175.0263
174.9516
87.0065
113.0565
131.0694
102.9547
118.3515
0
20
40
60
80
100
120
60
80
100
120
140
160
180
200

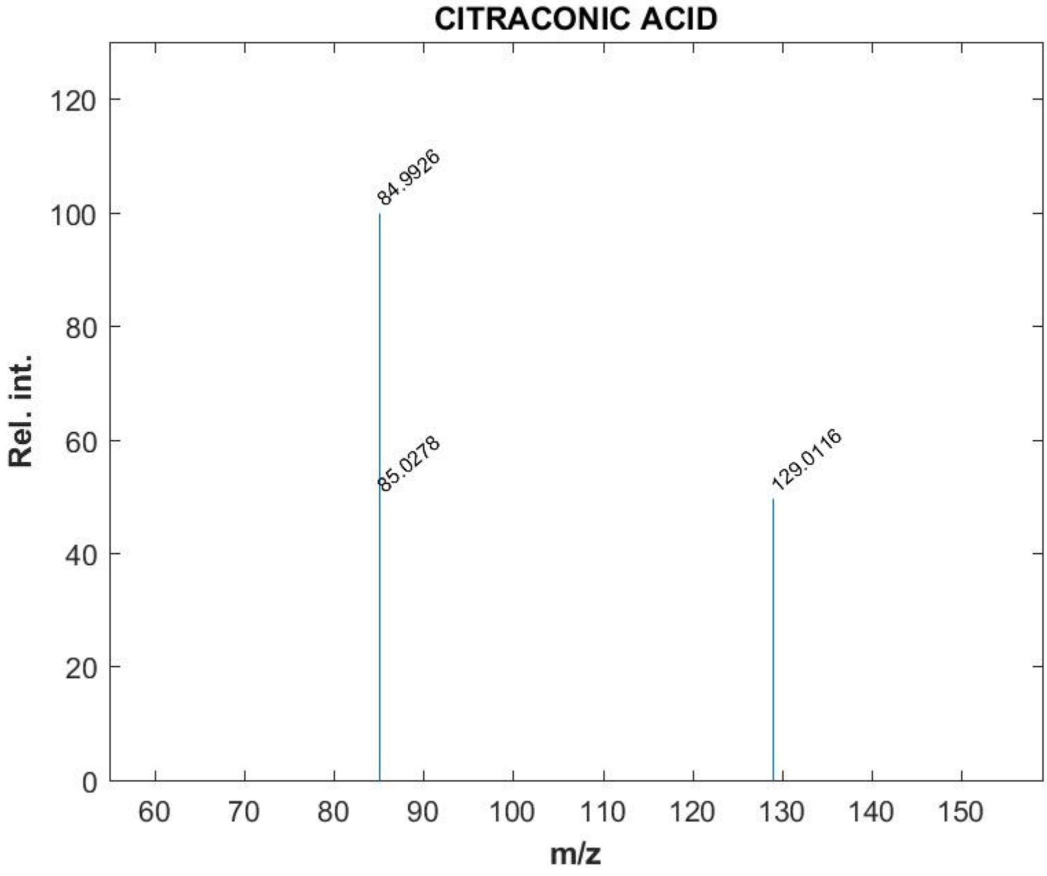
CITRACONIC ACID
Rel. int.
m/z
84.9926
85.0278
129.0116
0
20
40
60
80
100
120
60
70
80
90
100
110
120
130
140
150

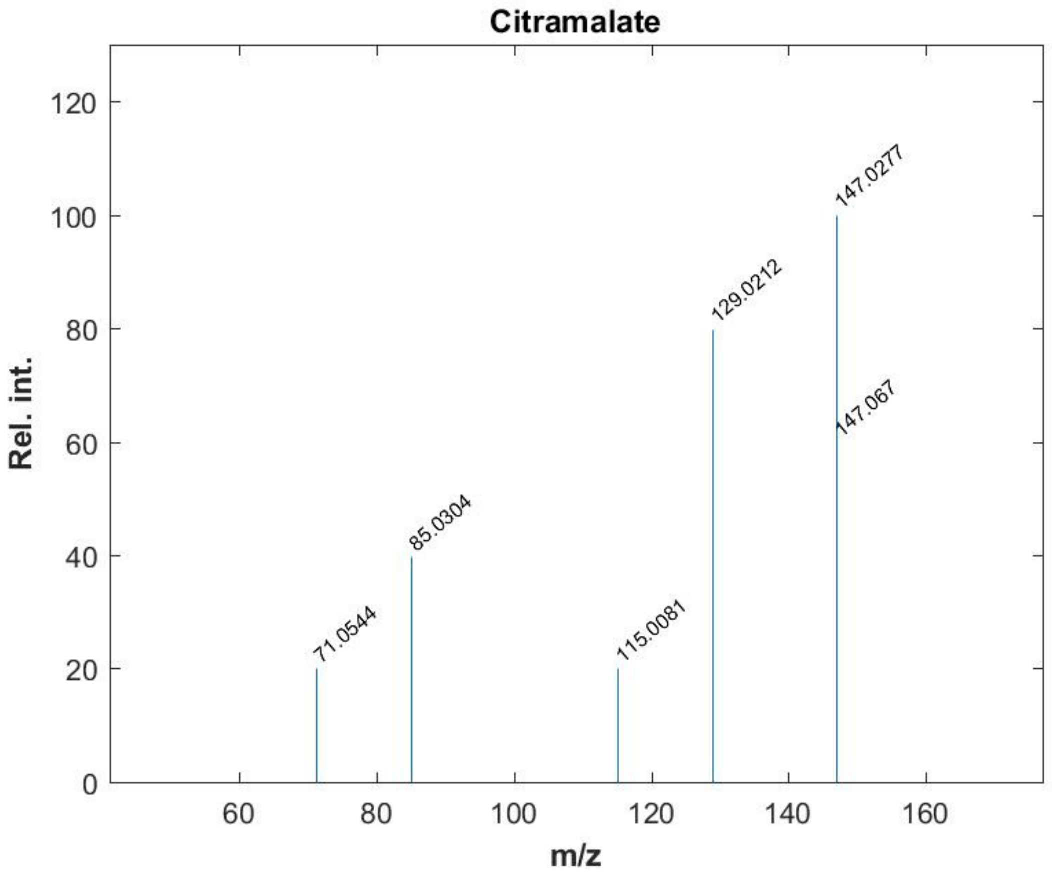

Citramalate
Rel. int.
m/z
0
20
40
60
80
100
120
60
80
100
120
140
160
71.0544
85.0304
115.0081
129.0212
147.0277
147.067

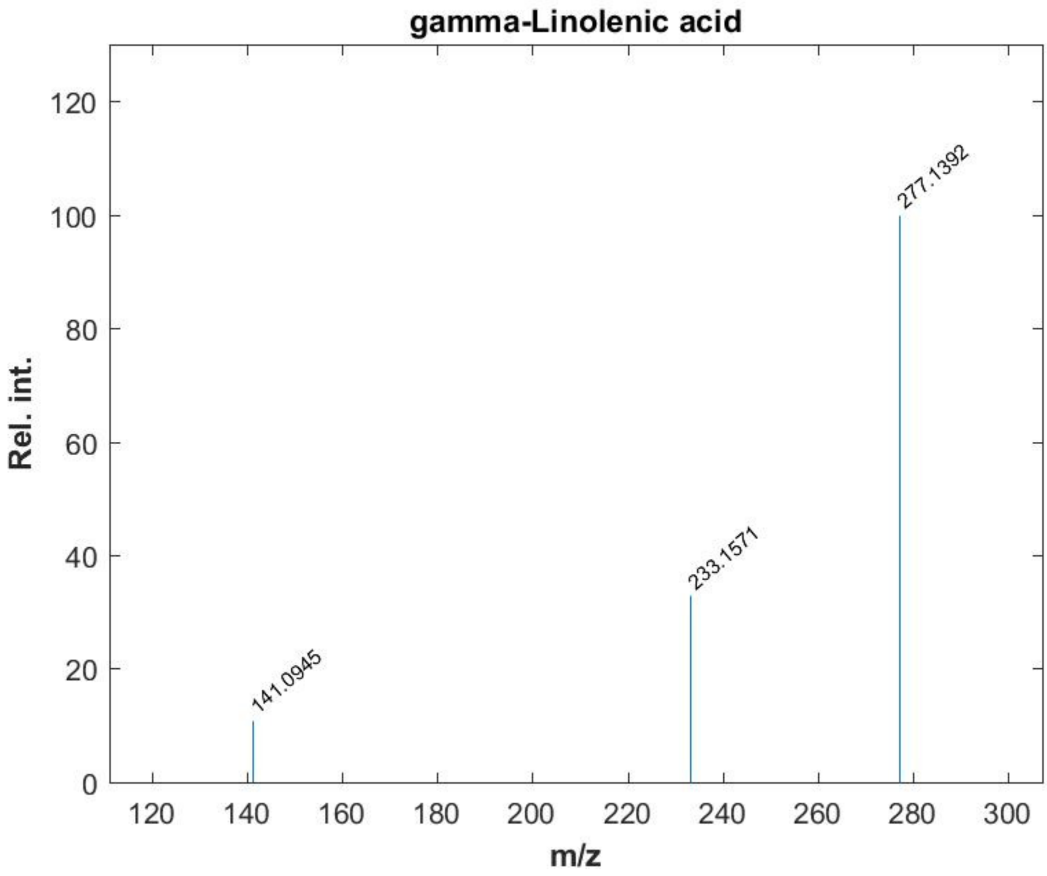
gamma-Linolenic acid
Rel. int.
m/z
0
20
40
60
80
100
120
120
140
160
180
200
220
240
260
280
300
141.0945
233.1571
277.1392

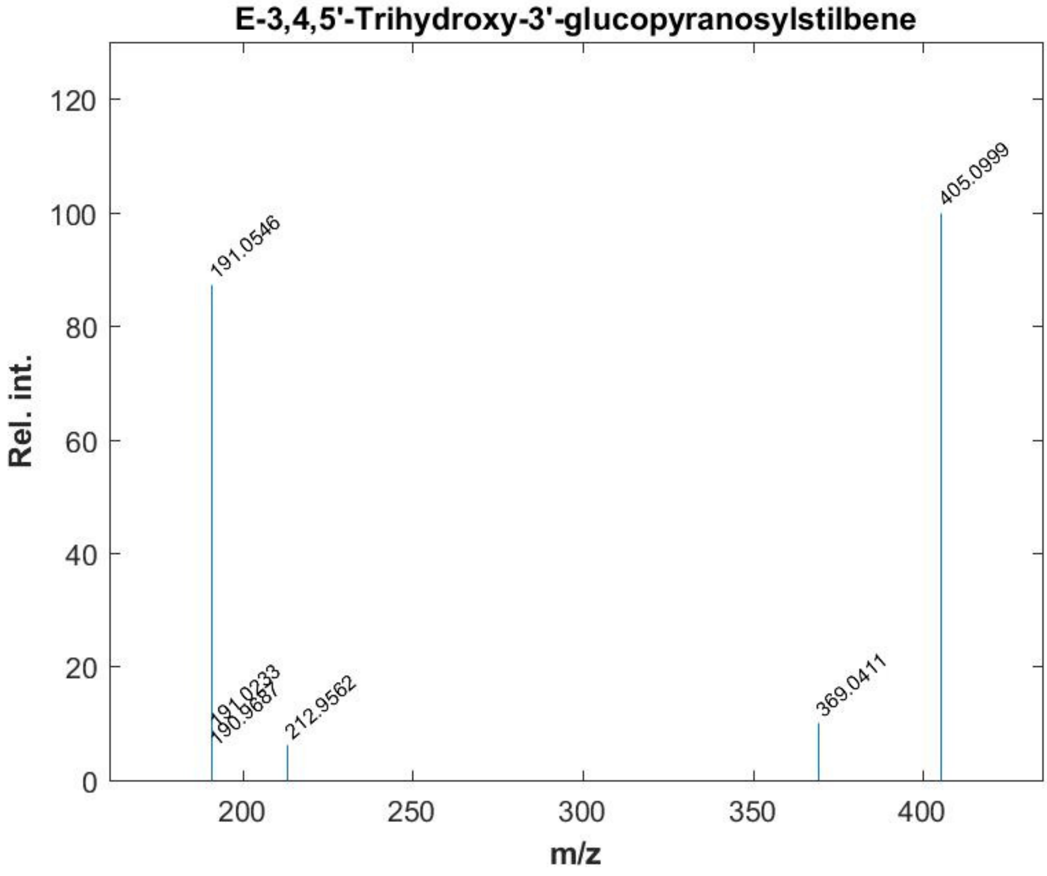
E-3,4,5'-Trihydroxy-3'-glucopyranosylstilbene
Rel. int.
m/z
0
20
40
60
80
100
120
200
250
300
350
400
191.0546
191.0233
190.9687
212.9562
369.0411
405.0999

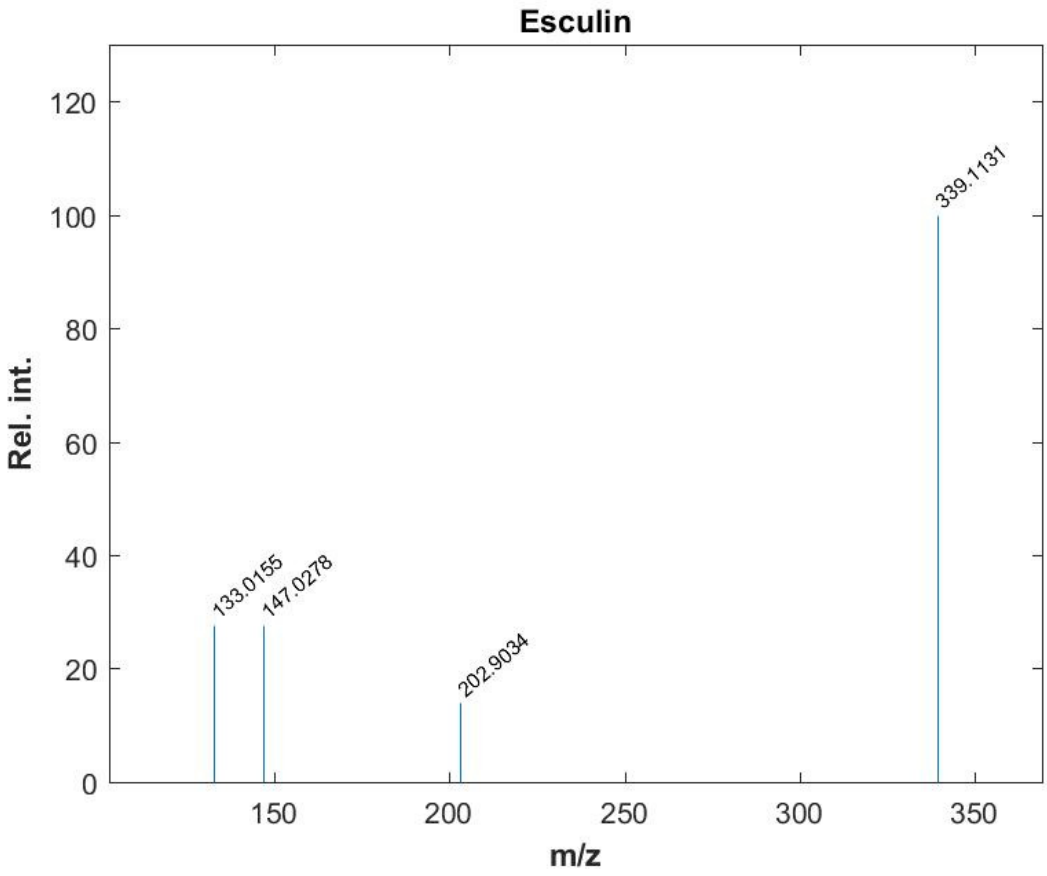
Esculin
339.1131
133.0155
147.0278
202.9034
Rel. int.
m/z
0
20
40
60
80
100
120
150
200
250
300
350

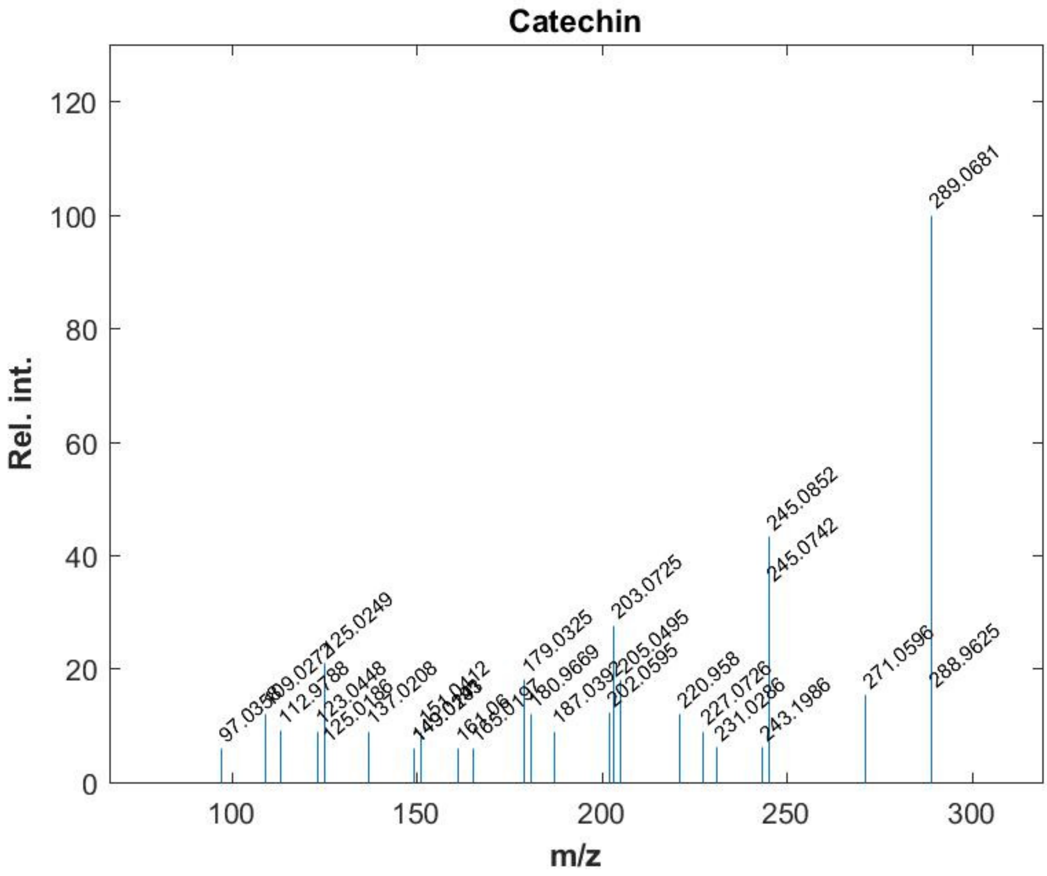
Catechin
Rel. int.
m/z
0
20
40
60
80
100
120
100
150
200
250
300
289.0681
245.0852
245.0742
203.0725
179.0325
125.0249
271.0596
288.9625
205.0495
202.0595
180.9669
187.0392
220.958
227.0726
231.0286
243.1986
97.0358
112.9788
123.0448
125.0186
137.0208
151.0412

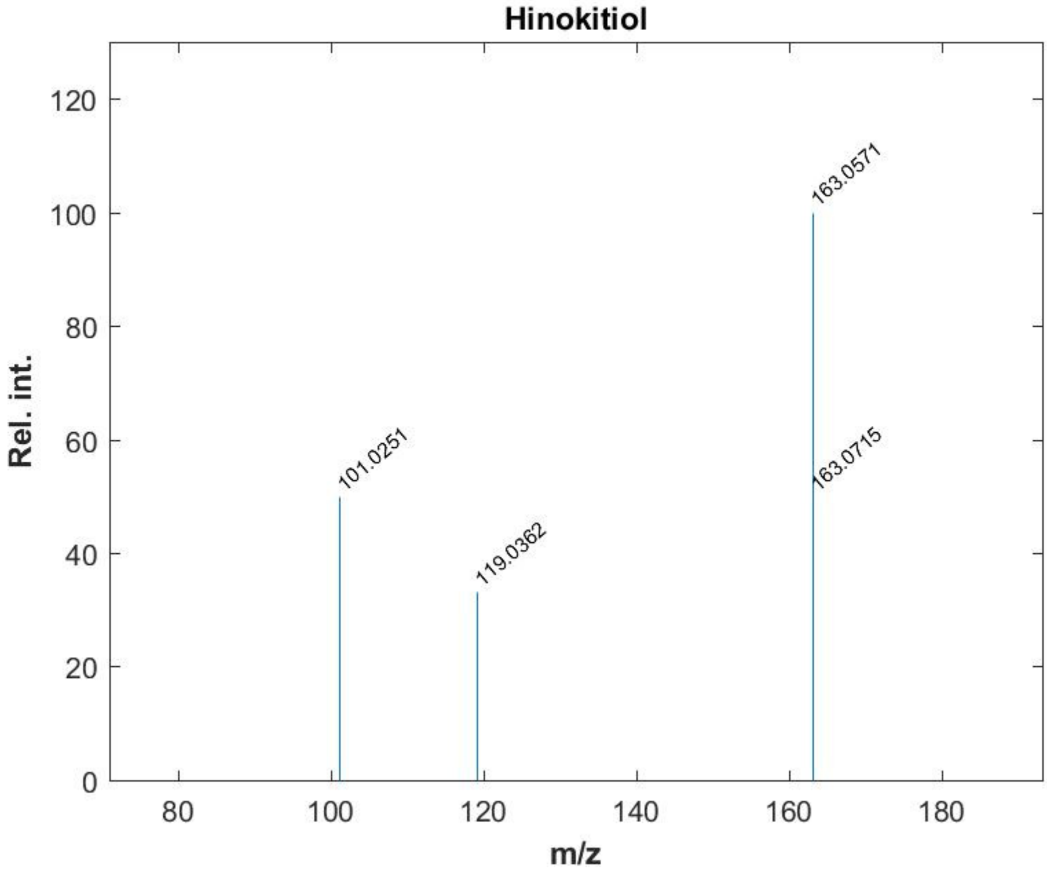
Hinokitiol
Rel. int.
m/z
0
20
40
60
80
100
120
80
100
120
140
160
180
163.0571
163.0715
101.0251
119.0362

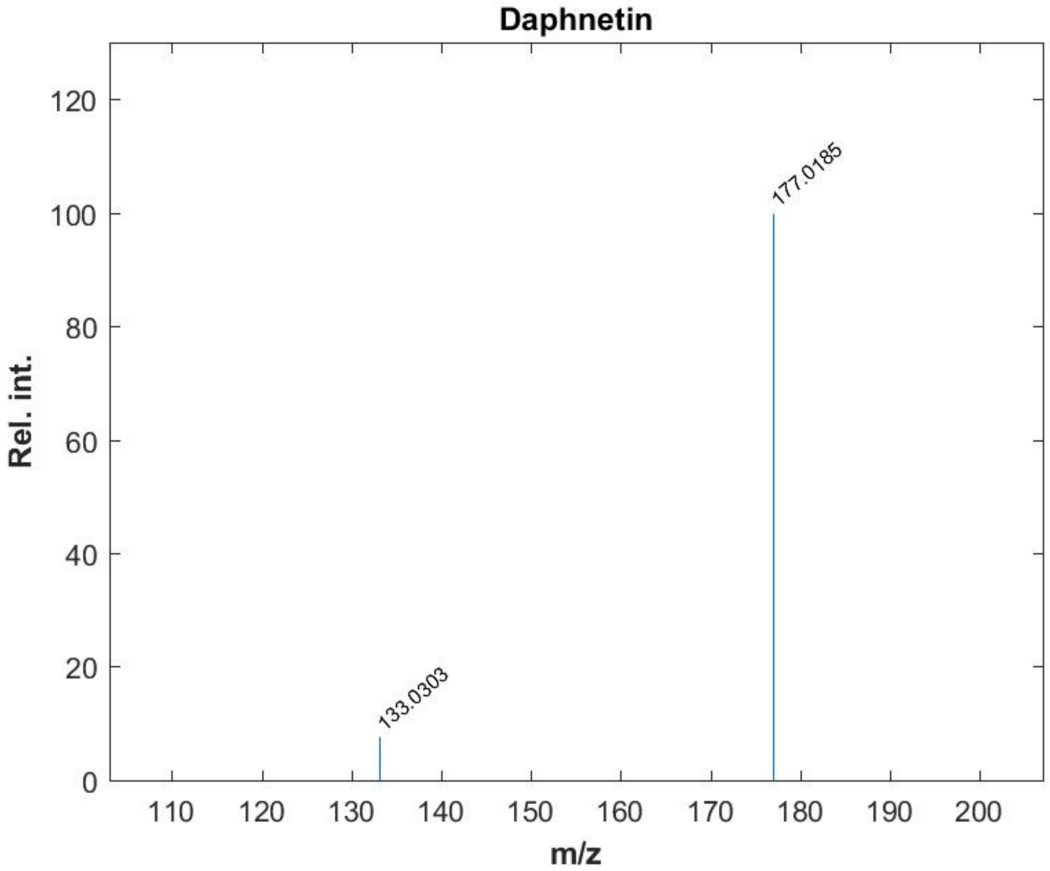
Daphnetin
Rel. int.
m/z
177.0185
133.0303
0
20
40
60
80
100
120
110
120
130
140
150
160
170
180
190
200

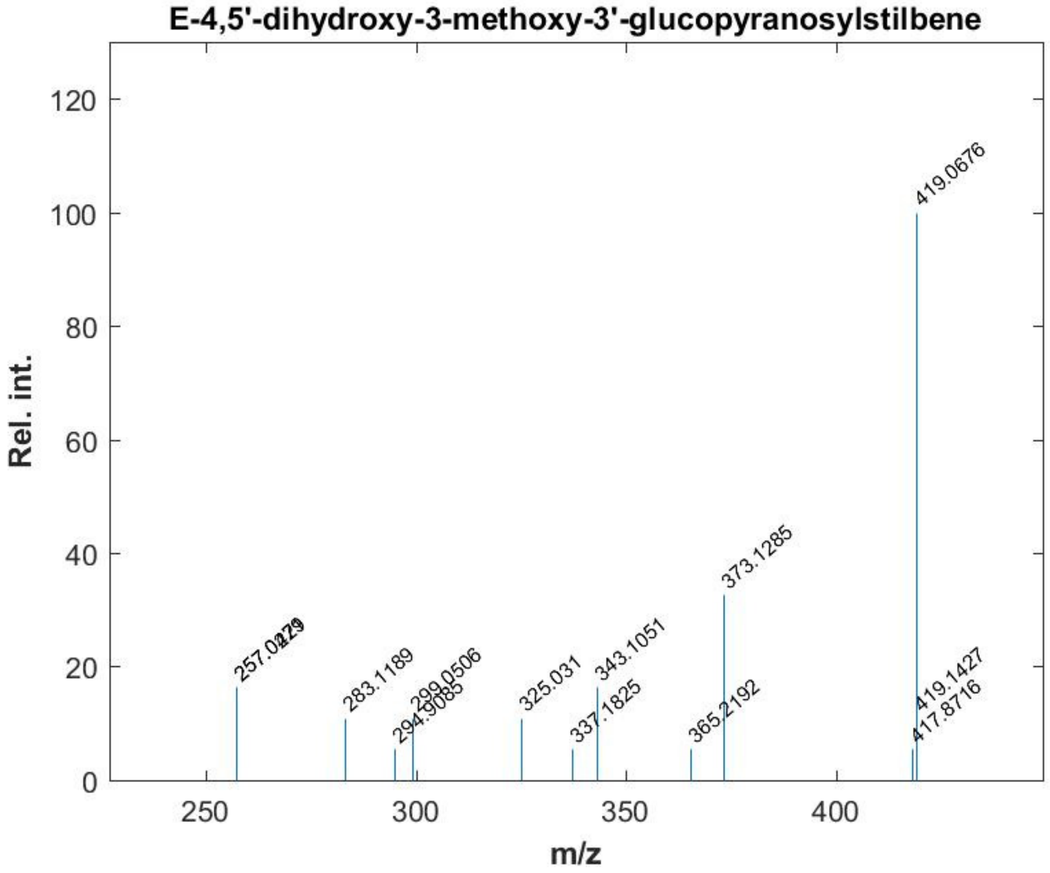
E-4,5'-dihydroxy-3-methoxy-3'-glucopyranosylstilbene
Rel. int.
m/z
419.0676
373.1285
343.1051
299.0506
283.1189
325.031
337.1825
365.2192
294.9085
419.1427
417.8716
257.0
0
20
40
60
80
100
120
250
300
350
400
